# Precise leaf damage detection across diverse species and environments via a large-scale vision model

**DOI:** 10.3389/fpls.2026.1788926

**Published:** 2026-03-26

**Authors:** Wei Chen, Hao Ruan, Peng Zhou, Lina He, Xinchao Ruan

**Affiliations:** 1 School of Information Science and Engineering, Xinjiang College of Science & Technology, Korla, China; 2 School of Environmental Engineering, Moutai Institute, Renhuai, China; 3 College of Physics and Electronic Information Engineering, Zhejiang Normal University, Jinhua, China

**Keywords:** deep learning paradigm, DinoV3, foundation model adaptation, interpretability analysis, leaf lesion segmentation, smart agriculture

## Abstract

Precise detection of crop leaf damage is essential for real-time plant health monitoring and yield estimation. However, conventional deep learning models often exhibit poor generalization when deployed across varying species and complex, unstructured field environments. To address these limitations, we propose a new modeling paradigm that shifts from traditional task-specific training to foundation model adaptation. Specifically, we introduce a novel architecture integrating the DinoV3 foundation model with a Unet framework to achieve robust leaf lesion segmentation. By incorporating a Spatial Prior Module (SPM) and a Projection Module, our approach effectively bridges the gap between general-purpose pre-training and domain-specific requirements. Experimental results on coffee and black gram datasets demonstrate that this paradigm consistently outperforms standard networks, including Unet, Unet++, and SwinUnet. On the coffee leaf dataset, the proposed model achieves an Intersection over Union (IoU) of 78.31% and a Pixel Accuracy of 88.00%, surpassing the baseline Unet by over 10.5% in IoU. Remarkably, the architecture reduces inference time by approximately 93.6% (from 63.41s to 4.07s), proving that high-parameter foundation models can be adapted for extreme computational efficiency in agricultural scenarios. To further validate scalability, we conduct additional experiments on a larger dataset, AMG*_HS_*. The proposed paradigm achieves the best overall detection performance while maintaining superior computational efficiency, confirming its robustness under increased data scale. Interpretability analysis reveals that the foundation model backbone effectively captures high-level semantic features of lesions, providing a clear explanation for its superior performance and cross-domain reliability. This research establishes a scalable, high-performance paradigm for intelligent crop protection, demonstrating that coupling customized encoders with foundation models is a superior strategy for cross-domain agricultural tasks.

## 1 Introduction

Accurate monitoring of plant diseases is crucial for ensuring crop yield and agricultural sustainability. Among various analysis tasks, leaf lesion segmentation plays a central role, as precise delineation of disease regions enables quantitative assessment of disease severity and progression. Extensive efforts have been devoted to the segmentation of plant organs and diseases. For instance, MC-Unet (Deng et al., 2023) delivers pixel-accurate delineation of tomato-leaf bacterial spot, late blight, early blight, and leaf mold under both clean and cluttered field conditions. To disentangle cucumber leaf lesions from heavily intertwined backgrounds, Yuan et al (Yuan et al., 2013). developed segmentation framework for cucumber diseases in complex scenes. Meanwhile, Panicle-SEG (Xiong et al., 2017) integrates deep learning with super-pixel refinement to achieve robust in-field segmentation of rice panicles. However, leaf lesion segmentation remains challenging in practice. Lesion regions often exhibit large variations in shape, scale, and visual appearance across different plant species and disease types. Moreover, lesion boundaries are frequently diffuse and ambiguous, making it difficult to distinguish diseased tissue from healthy regions at the pixel level (Shafay et al., 2025; Salka et al., 2025). These challenges are further compounded by the high cost and labor-intensive nature of acquiring dense, pixel-wise annotations in agricultural settings, which limits the availability of large-scale labeled datasets.

Recent advances in leaf lesion segmentation have increasingly explored complex architectures, including Transformer-based and hybrid convolution-attention models, in an attempt to capture long-range dependencies and rich contextual information. AISOA-SSformer (Dai et al., 2024) proposes a Transformer-based semantic segmentation framework that integrates sparse global-update learning, salient feature attention, and annealing-integrated sparrow optimization to accurately segment rice leaf disease regions under complex field conditions. SegFormer (Xie et al., 2021) introduces a lightweight Transformer-based semantic segmentation framework that employs a hierarchical Transformer encoder with multi-scale feature extraction and a simple MLP decoder to efficiently fuse local and global contextual information for accurate semantic segmentation. LSMAE (Liu et al., 2022a) proposes a self-supervised Transformer-based pre-training framework that combines k-NN guided feature relationship conditional filtering with latent semantic masking auto-encoding to learn discriminative representations for accurate pest and disease classification across domains. GeT (Lu et al., 2022) presents a lightweight hybrid diagnosis framework that integrates Ghost convolution with Transformer encoders to efficiently capture global contextual information for accurate and real-time grape leaf disease and pest recognition in complex field environments. DICNN (Liu et al., 2020) proposes an improved convolutional neural network that incorporates Inception-based multi-dimensional feature extraction and dense connectivity to enable accurate recognition of grape leaf diseases from large-scale augmented field image datasets. An improved YOLOv3-based method (Liu and Wang, 2020) employs image pyramid-enhanced multi-scale feature learning to enable fast and accurate detection and classification of tomato diseases and pests in real-world natural environments. DCCAM-MRNet (Liu et al., 2022b) proposes a noise-robust tomato leaf disease recognition framework that integrates INLM filtering, dilated convolution, coordinate attention, and mixed residual connections to accurately detect small and inconspicuous disease spots in complex field environments. A cross-modal segmentation network (CM-Net) (Wang et al., 2024) is proposed for winter wheat mapping by integrating multi-temporal remote-sensing imagery and DEM data. The network incorporates a diverse receptive fusion module for scale-adaptive feature fusion and a distributed weight attention module to enhance discriminative feature representation during decoding. Sen Liu et al (Liu et al., 2025). propose a hybrid decision-making framework that combines three-way decision theory with the DEA game cross-efficiency method to support the selection of order procurement modes in contract farming. The method determines decision-maker weights using T-spherical fuzzy sets and cosine similarity, constructs an evaluation matrix and loss function, and sets probability thresholds based on game cross-efficiency, with its effectiveness validated through multiple case studies. Lulu li et al (Li et al., 2024). propose the Grasping with Occlusion-Aware aLly (GOAL) method based on binocular stereo vision for robotic arm grasping. The method first infers occlusion relationships to segment and localize multiple targets, and then performs multi-target grasping pose estimation to determine effective grasping positions. Tao Sun et al (Sun et al., 2025). propose RID-LIO, an intensity-assisted LiDAR-inertial SLAM framework that integrates adaptive intensity feature extraction and intensity-based loop detection. The method projects 3D point clouds to intensity images to extract line features, applies a weighting function for pose optimization, and uses an intensity edge context descriptor to improve loop detection and reduce trajectory drift. While these designs offer greater modeling capacity, they also introduce substantially more parameters and weaker inductive biases compared to convolutional architectures. As a result, such models typically require large amounts of annotated data to learn stable and meaningful representations. In agricultural imaging, where lesion appearances vary significantly across species, growth stages, and environmental conditions, limited training data often leads these high-capacity models to overfit to dataset-specific patterns rather than learning transferable visual features. Consequently, their performance may degrade when applied to new datasets, disease types, or imaging conditions, limiting their generalization ability in practical agricultural scenarios.

Foundation models refer to large-scale neural networks pretrained on massive and diverse datasets using self-supervised or weakly supervised objectives, with the goal of learning general-purpose visual representations that can be transferred across tasks and domains (Qin et al., 2024; Pai et al., 2024). Unlike task-specific models trained from scratch, foundation models emphasize representation learning at scale, enabling them to capture rich semantic structures and robust visual patterns without relying on dense annotations. The key advantage of foundation models lies in their ability to leverage large-scale pretraining to reduce dependence on labeled data while improving generalization. Through self-supervised learning, these models acquire strong inductive biases from data diversity rather than architectural constraints (Xue et al., 2025; Zhang et al., 2024; Harris et al., 2025; Gao et al., 2025), making their learned representations more stable and transferable under limited supervision. Such properties have led to substantial success in both natural image understanding and medical image analysis. In natural images, foundation models demonstrate strong transferability across diverse visual tasks and datasets. Mask Dino (Li et al., 2023) leverages a foundation detection model (Dino) with shared query embeddings to unify object detection and instance, panoptic, and semantic segmentation within a single scalable framework. Dino-WM (Zhou et al., 2024) leverages foundation visual features pre-trained with Dinov2 to model visual dynamics from offline trajectories, enabling task-agnostic planning and zero-shot behavioral prediction across diverse environments without task-specific supervision. In medical imaging, where annotations are scarce and visual patterns can be subtle and heterogeneous, pretrained foundation models have been shown to improve segmentation and recognition performance by providing robust feature representations (He et al., 2023). employs a foundation segmentation model to provide zero-shot mask predictions across diverse medical imaging datasets (Cheng et al., 2023). achieved high-quality segmentation across public medical datasets covering various organs and imaging modalities with the SAM model, using only a handful of prompts—just a few points or bounding boxes.

Agricultural vision faces distinct hurdles that parallel these domains, particularly the high intra-class variability across diverse species and the unstructured nature of field environments (da Silva et al., 2025; Jiang et al., 2024). While foundation models have shown promise in general tasks, their capacity to handle the fine-grained nuances of agricultural lesions—especially when transitioning across different crops—remains largely underexplored. This gap suggests that adapting foundation model representations could provide the robust generalization necessary for reliable disease analysis in fluctuating outdoor conditions.

Taken together, these observations highlight a critical oversight in current agricultural lesion segmentation research. Prevailing methodologies often gravitate toward increasingly intricate decoder architectures to enhance performance. However, this trend implicitly assumes that structural complexity is the primary driver of accuracy, leaving the pivotal role of representation quality—and its direct impact on high-precision diagnostic outcomes—largely underexplored within the agricultural context. Motivated by this, we investigate a representation-first design aimed at achieving superior precision in leaf lesion segmentation. Rather than escalating decoder complexity, our approach prioritizes the enrichment of visual features through large-scale self-supervised pretraining. Specifically, we leverage Dinov3 as a foundation-model encoder to extract semantically dense and highly transferable representations. These robust features enable the model to discern fine-grained necrotic patterns with exceptional clarity, which are then mapped by a streamlined Unet decoder to produce high-fidelity segmentation masks. Our experimental findings consistently reveal that segmentation precision is governed more by the quality of learned representations than by the sophistication of the decoder. Notably, under data-constrained conditions, a standard Unet decoder paired with robust foundation-model features achieves competitive, and often superior, accuracy compared to state-of-the-art complex architectures. This shift in focus underscores the practical value of representation-first modeling for advancing precise and reliable agricultural disease diagnostics.

The primary contributions of this work are threefold:

(1) Compared to traditional Unet methods, our approach achieves superior diagnostic accuracy across multiple species and environments. This validates its exceptional robustness in transitioning from controlled settings to complex, unstructured field conditions.
(2) By incorporating a Spatial Prior Module (SPM) and a Projection Module, the model effectively preserves spatial hierarchies and aligns foundation-model features. This integration ensures the high-fidelity segmentation of subtle and irregular necrotic patterns.
(3) The framework reduces inference time by 93.6% compared to Unet, facilitating real-time monitoring. Furthermore, interpretability analysis of feature representations across different models confirms the theoretical and practical feasibility of our approach.
(4) This work establishes a representation-first paradigm, simplifying the modeling process. This 151 shift provides a scalable and efficient strategy for advancing precise crop health diagnostics.

## 2 Materials and methods

### 2.1 Dataset and annotation

**Figure 1:**
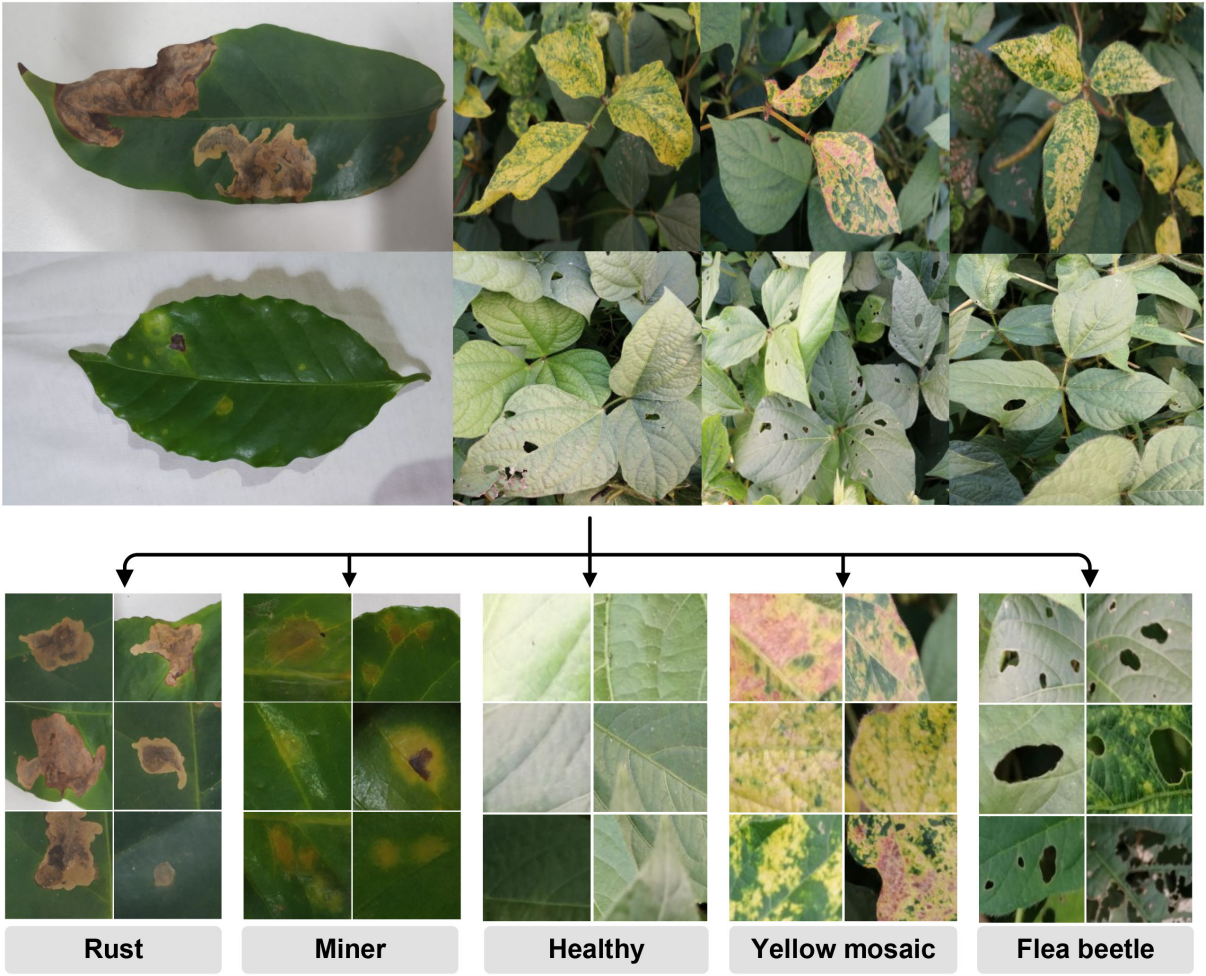
Example images of the pest and disease damage dataset. Samples include coffee leaves (laboratory setting) and black gram leaves (field setting). The dataset categorizes four types of damage: Rust, Miner, Yellow Mosaic, and Flea Beetle.

As shown in **Figure 1**. We utilize different leaf disease datasets to evaluate our representation-first segmentation approach, selected to cover a range of imaging environments and lesion complexities.

Coffee leaf dataset (Silva et al., 2020): (simple background): This dataset contains images of coffee leaves collected from a farm in Brazil, annotated manually for rust (Hemileia) and leaf miner damage. The dataset includes 285 rust images and 257 leaf miner images, captured using a smartphone at 4000×2250 resolution in RGB format. Most images were taken in a laboratory setting against a white background, providing a controlled environment where each leaf and lesion is clearly visible. The rust lesions appear as orange powdery patches on the upper leaf surface, whereas leaf miner damage is characterized by serpentine tunnels caused by larval feeding. This dataset allows testing the model under simple conditions where the background does not introduce visual clutter, serving as a baseline to evaluate the ability of the representation encoder to detect clear, isolated lesions.

Black gram dataset (Hridoy et al., 2025) (complex background): Collected in Southeast Asia under real-field conditions, this dataset contains 587 high-resolution images (1024x1024) of black gram leaves, annotated for three lesion types: LA (healthy leaves), LB (flea beetle damage), and LC (yellow mosaic). Images often include multiple overlapping leaves, multiple lesions per leaf, variable lighting, shadows, and other field-level visual complexities such as soil background and partial occlusions. LA represents normal leaf tissue, LB lesions are small, round feeding marks caused by flea beetles, and LC lesions are irregular yellow patches associated with yellow mosaic disease. Using this dataset evaluates the model’s robustness under real-world conditions, where the representation encoder must disentangle leaf structure, background clutter, and multiple lesion appearances, highlighting the importance of strong learned representations rather than just architectural sophistication.

The AMG*_HS_*dataset (Sama et al., 2023) is an extended version of the AMG dataset, meticulously curated to identify and highlight instances of plant stress in crop images. It comprises 6,127 RGB images, each with a resolution of 120 × 120 pixels, carefully selected from the AMG dataset, which encompasses multiple plant species. The dataset is divided into healthy samples (3,798 images) and stressed samples (2,329 images), covering 14 out of the 18 categories in the AMG dataset. In addition to classification labels for distinguishing between healthy and stressed conditions, the dataset also provides segmentation masks for precise localization of stress-affected regions.

**Figure 2:**
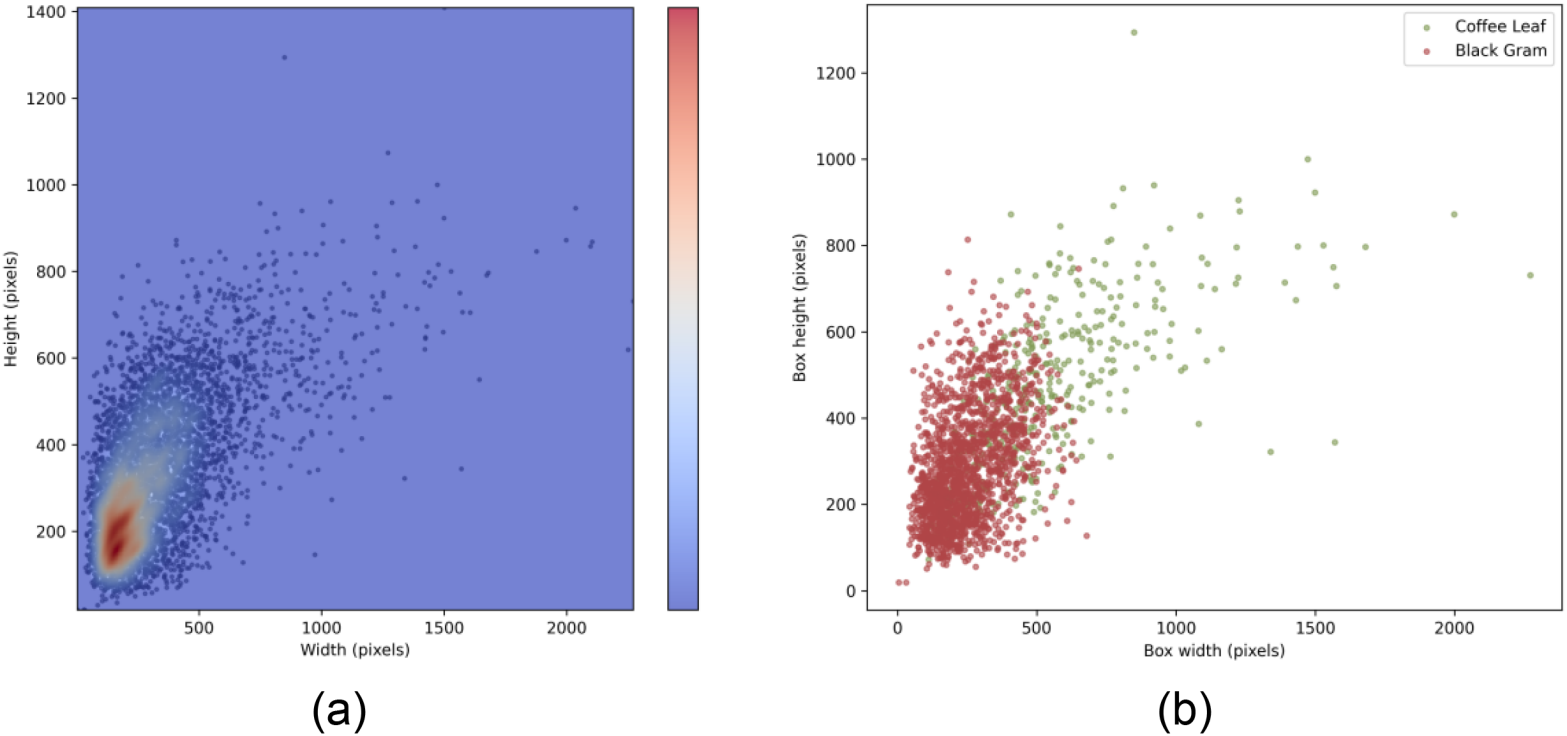
Comparison of pixel distributions for objects of different sizes across: **(A)** the entire dataset and **(B)** the test subset.

**Figure 2** illustrates the distribution of damage scales within the collected real-world dataset, specifically detailing the varied lesion sizes across different crop species in the test set. **Table 1** summarizes the datasets, subgroups, lesion types, image counts, annotation details, and environmental conditions, highlighting the diversity in data complexity and acquisition settings.

**Table 1:** Comprehensive overview of the datasets.

Dataset	Subset	Images	Lesion types	Image resolution	Annotation type	Notes
Black gram	Train	412	LA/LB/LC	1024×1024	Bounding box (manual)	Outdoor, complex background
Black gram	Val	59	LA/LB/LC	1024×1024	Bounding box (manual)	Outdoor, complex background
Black gram	Test	116	LA/LB/LC	1024×1024	Bounding box (manual)	Outdoor, complex background
Coffee leaves	Train	379	Rust/Leaf miner	4000×2250	Manual annotation	Indoor lab, white background, high resolution
Coffee leaves	Val	55	Rust/Leaf miner	4000×2250	Manual annotation	Indoor lab, white background, high resolution
Coffee leaves	Test	108	Rust/Leaf miner	4000×2250	Manual annotation	Indoor lab, white background, high resolution
AMG*_HS_*	Train	4288	Health/Stress	512×512	Semantics segmentation	Outdoor, complex background
AMG*_HS_*	Val	612	Health/Stress	512×512	Semantics segmentation	Outdoor, complex background
AMG*_HS_*	Test	1225	Health/Stress	512×512	Semantics segmentation	Outdoor, complex background

Split ratios are Train: Val : Test = 7:1:2. LA: Healthy leaves; LB: Flea beetle damage; LC: Yellow mosaic disease.

### 2.2 Experimental setup and data splitting

All experiments were conducted on a workstation equipped with an NVIDIA RTX 3090 GPU (24 GB VRAM), an Intel x86_64 CPU with 16 cores, and 64 GB of system memory. Both datasets were split into training, validation, and test subsets at the image level to prevent data leakage between sets. Both datasets adopted an identical 7:1:2 train-val-test split. This setup ensures a fair evaluation of model performance across both simple and complex imaging environments. Images were resized to 256 × 256 pixels using torchvision.transforms. Resize before being loaded into the DataLoader. All images were preprocessed consistently to maintain input resolution and normalized before training. The uniform experimental setup allows a direct comparison of segmentation performance and highlights the role of representation quality independent of hardware or preprocessing differences.

### 2.3 Model architecture

#### 2.3.1 The introduction of DinoV3

We adopt Dinov3 as the backbone encoder for leaf lesion segmentation. Dinov3 is a self-supervised visual foundation model developed by Meta, integrating ViT and ConvNeXt architectures. It is pre-trained on large-scale unlabeled datasets, including LVD-1689M, using self-distillation and masked modeling strategies, which produce high-quality dense feature representations suitable for zero and few-shot learning.

In our workflow, each input leaf image *x* is first passed through the Dinov3 encoder to extract dense feature maps *f*_encoder_(*x*). These features capture rich semantic and structural information at multiple spatial scales, which are critical for segmenting lesions that are small, irregular, or have low contrast with the background. Formally, the encoder outputs can be represented as Equation 1:

(1) $$f_{\text{encoder}}(x)=\text{Dinov}3(x),\;f_{\text{encoder}}(x)\in {\mathbb{R}}^{H^{\prime }\times W^{\prime }\times C}$$

where $H^{\prime }$ and $W^{\prime }$ denote the spatial dimensions and C is the channel dimension of the feature map.

#### 2.3.2 DinoUnet

**Figure 3:**
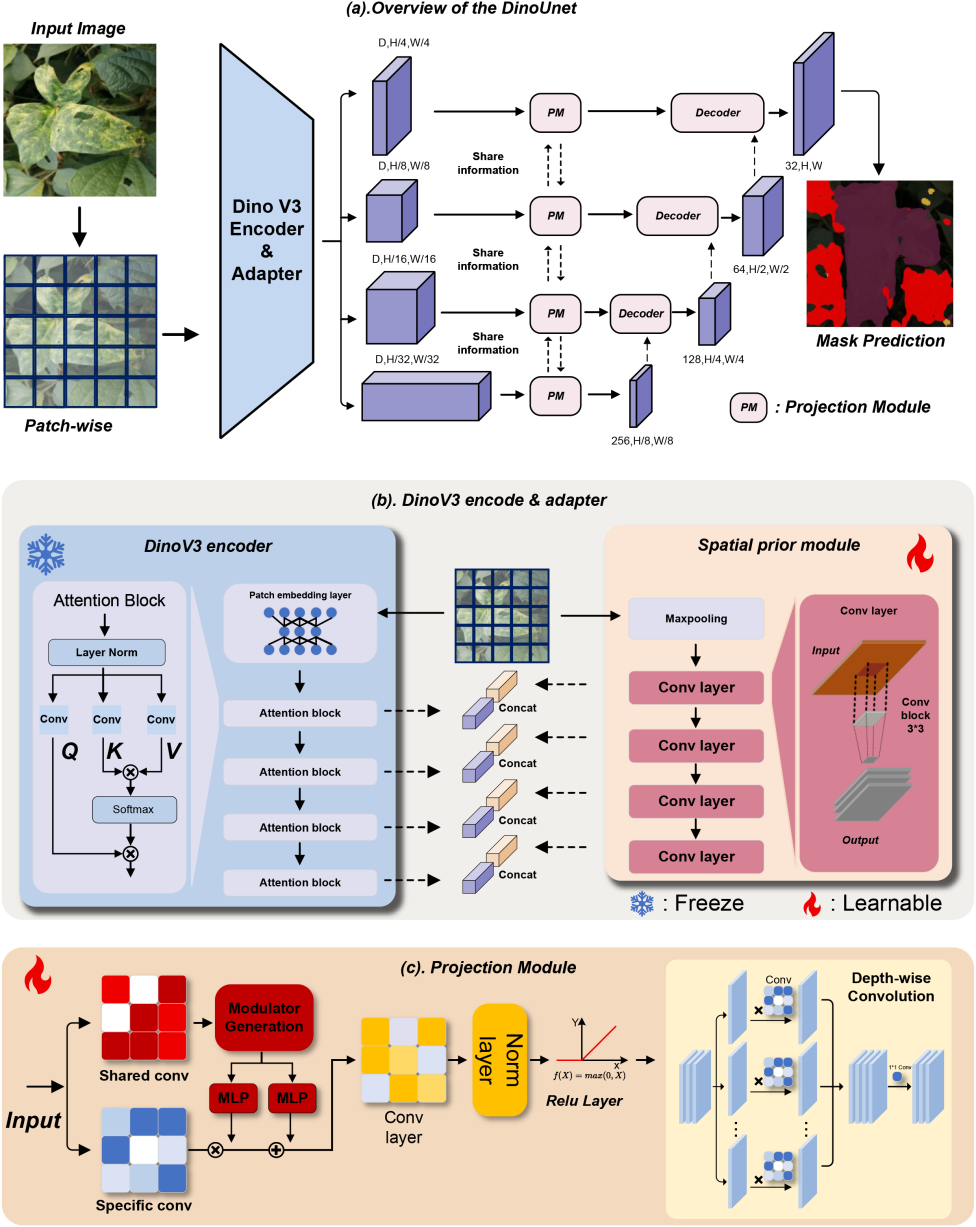
Architecture of the proposed DinoUnet model. **(A)** Overview of the DinoUnet framework; **(B)** Illustration of the internal workflow, highlighting the DinoV3 encoder and the Spatial Prior Module; **(C)** Structural details of the Projection Module.

To further improve leaf lesion segmentation, we propose DinoUnet, an encoder-decoder architecture that integrates a Dinov3 encoder with a Spatial Prior Module and a Projection Module. The core design of DinoUnet lies in effectively fusing high-level semantic representations from Dinov3 with low-level spatial details, while ensuring feature compatibility with the decoder through efficient projection. The detailed structure is illustrated in **Figure 3**.

To complement the semantic-focused representations from Dinov3, the SPM captures fine-grained spatial information, such as lesion edges, local texture variations, and subtle shape details, which are critical for distinguishing small or ambiguous lesions from the background. The SPM processes the input leaf image *x* through a set of lightweight convolutional layers with progressive downsampling, generating multi-scale spatial feature maps *f*_spm_(*x*) aligned with the spatial resolution of the encoder features *f*encoder(*x*). The detailed formulation is presented in Equation 2:

(2) $$\begin{array}{l} f_{\text{spm}}(x)=(\prod_{i=1}^{4}{\text{ConvBlock}}_{i})\circ \text{MaxPool}(x),\;f_{\text{spm}}(x)\\ \in {\mathbb{R}}^{H^{\prime }\times W^{\prime }\times C_{\text{spm}}} \end{array}$$

where *C*_spm_ denotes the channel dimension of the spatial feature maps, and $H^{\prime }$ and $W^{\prime }$ are the spatial dimensions compatible with *f*_encoder_(*x*) to enable direct feature fusion. MaxPool(·) denotes the initial downsampling layer, and ${\left\{{\text{ConvBlock}}_{i}\right\}}_{i=1}^{4}$ represents the sequence of four lightweight convolutional layers designed to capture fine-grained spatial details.

The semantic features from Dinov3 and the spatial features from SPM are then integrated via a cross-attention-based fusion mechanism. This operation dynamically aligns and aggregates complementary information, producing enriched multi-modal features *f*_fuse_(*x*). (The computation is formally defined in Equation 3) that combine semantic discriminability with spatial precision:

(3) $$f_{\text{fuse}}(x)=F_{\text{fusion}}(f_{\text{encoder}}(x),f_{\text{spm}}(x))$$

where $F_{\text{fusion}}$ denotes the concatenation operation for feature fusion.

To reconcile the channel dimension mismatch between the fused features *f*_fuse_(*x*) and the Unet decoder, the Projection Module (PM) performs efficient dimensionality reduction while preserving high-fidelity details. It maps the fused features to a target channel dimension *C*_target_ suitable for the decoder, producing the projected features *f*_proj_(*x*), the calculation is described in Equation 4:

(4) $$f_{\text{proj}}(x)=\text{PM}(f_{\text{fuse}}(x)),\;f_{\text{proj}}(x)\in {\mathbb{R}}^{H^{\prime }\times W^{\prime }\times C_{\text{target}}}$$

Finally, the Unet decoder receives *f*_proj_(*x*) as skip connections. Through iterative upsampling and feature refinement, it generates the final pixel-wise segmentation mask *M* for leaf lesions, the computation process is described in Equation 5:

(5) $$M=D_{\text{Unet}}(f_{\text{proj}}(x))$$

This design efficiently leverages strong pre-trained Dinov3 features while incorporating spatially precise details, enabling accurate segmentation even under small-sample and high-variability scenarios typical of agricultural leaf lesion datasets.

#### 2.3.3 Methods of comparative approaches

To ensure a systematic and progressive comparison, we selected Unet, Unet++, Swin-Unet, and DinoUnet as representative models with gradually enhanced modeling capabilities. Specifically, Unet, as a classical encoder–decoder convolutional neural network architecture, represents the local modeling paradigm based on fully convolutional networks (FCNs). Unet++ extends Unet by introducing nested and dense skip connections, aiming to reduce the semantic gap between encoder and decoder feature maps and to enhance multi-scale feature fusion. SwinUnet is built upon a hierarchical Transformer backbone, replacing conventional convolution-based feature extraction with a self-attention mechanism, thereby enabling the modeling of long-range dependencies and global contextual information. Finally, DinoUnet incorporates a self-supervised pretrained foundation model (a Dino-pretrained Vision Transformer) into the segmentation framework, leveraging large-scale pretraining knowledge to improve feature representation capability and model generalization performance. The primary motivation for selecting these approaches is to systematically analyze how the introduction of a foundation model influences segmentation performance and representation capacity, in comparison with the three original modeling paradigms: convolution-based baseline models, multi-scale convolution-enhanced models, and Transformer-based global modeling architectures.

Unet (Ronneberger et al., 2015) is a classical encoder-decoder network with a symmetric U-shaped architecture. Its simple convolutional design and skip connections allow the decoder to recover spatial details lost during downsampling. Despite its relatively small parameter count and robustness in low-data regimes, its performance may be limited by the representational capacity of the learned features.

Unet++ (Zhou et al., 2019) extends Unet with nested dense skip pathways, aggregating features across multiple encoder layers to reduce the semantic gap between encoder and decoder. Deep supervision enables multi-level outputs, improving boundary precision, especially for fine or irregular structures. While these modifications enhance segmentation accuracy, they also increase network depth and computational cost.

SwinUnet (Liu et al., 2021), a fully Transformer-based U-shaped network, replaces convolutions with Swin Transformer blocks. By capturing both local and global dependencies through windowed self-attention and shifted windows, it can model complex shapes and low-contrast regions effectively. Multi-scale skip connections preserve spatial information in the decoder. Although Swin-Unet provides stronger representational capacity than CNN-based baselines, it also entails higher computational complexity and larger memory requirements.

### 2.4 Training strategy and implementation details

The model is trained using a combination of Cross-Entropy (CE) Loss and Focal Loss to handle class imbalance and improve segmentation of small or sparse lesions. The CE Loss penalizes incorrect predictions at the pixel level. For a single pixel with true class *y* ∈ {0,1} and predicted probability $\hat{p}$, the CE Loss is defined as shown in Equation 6:

(6) $$L_{CE}=-\sum_{c=1}^{C}y_{c}\log\;({\hat{p}}_{c})$$

where *C* is the number of classes and *y_c_*is the one-hot encoded true label.

The Focal Loss adds a modulating factor to focus learning on hard-to-classify pixels, the formulation is presented in Equation 7:

(7) $$L_{FL}=-\sum_{c=1}^{C}\alpha_{c}(1-{\hat{p}}_{c}{)}^{\gamma }y_{c}\log\;({\hat{p}}_{c})$$

where *α_c_*is a class weighting factor, and *γ* is a focusing parameter that reduces the loss contribution from well-classified pixels ( ${\hat{p}}_{c}\to 1$). Focal Loss is particularly effective for small, sparse, or difficult to-detect lesions, as it emphasizes harder examples during training.

The combined loss used for training is defined as shown in Equation 8:

(8) $$L_{total}=L_{CE}+L_{FL}$$

This combination ensures accurate pixel-level classification while improving the detection and segmentation of challenging lesion regions.

### 2.5 Evaluation metrics

To comprehensively evaluate the segmentation performance, we used a set of widely adopted metrics that capture both pixel-level accuracy and mask overlap quality.

Dice coefficient (Dice): Measures the overlap between the predicted mask and the ground truth. It is particularly sensitive to small or sparse lesions, making it suitable for evaluating fine-grained leaf disease segmentation. The specific computation is shown in Equation 9.

(9) $$\text{Dice}=\frac{2\;|P\cap G|}{\left|P|+|G\right|}$$

where *P* is the set of predicted lesion pixels and *G* is the set of ground-truth lesion pixels.

Intersection over Union (IoU): Also known as the Jaccard Index, it quantifies the proportion of overlap relative to the union of prediction and ground truth. IoU complements Dice by penalizing both false positives and false negatives. The calculation is described in Equation 10.

(10) $$\text{IoU}=\frac{\left|P\cap G\right|}{\left|P\cup G\right|}$$

Pixel Accuracy (PA): Computes the ratio of correctly classified pixels to the total number of pixels. PA provides a straightforward measure of overall segmentation correctness but may be less sensitive to class imbalance. The detailed computation is given in Equation 11.

(11) $$\text{PA}=\frac{\text{Number of correctly classified pixels}}{\text{Total number of pixels}}$$

Precision, Recall, and F1 Score: These metrics assess the model’s ability to correctly identify lesion pixels. Equations 12–14 present their respective computational formulas.

(12) $$\text{Precision}=\frac{TP}{TP+FP}$$

(13) $$\text{Recall}=\frac{TP}{TP+FN}$$

(14) $$\text{F}1=\frac{2\cdot \text{Precision}\cdot \text{Recall}}{\text{Precision}+\text{Recall}}$$

where *TP*, *FP*, and *FN* are true positive, false positive, and false negative pixel counts, respectively. Precision measures how many predicted lesion pixels are actually correct. Recall evaluates how many true lesion pixels are successfully detected. F1 Score balances precision and recall, providing a single measure of segmentation quality. Together, these metrics provide a comprehensive evaluation, capturing both overall accuracy and the model’s ability to detect small or challenging lesions, which is critical in leaf disease segmentation tasks.

### 2.6 Model interpretability analysis

We conducted embedding feature visualization to interpret the model’s decision-making and assessed correlations between embeddings and evaluation metrics, aiming to evaluate the efficiency of different foundation-based modeling approaches.

To investigate the representational capacity of the encoder features, we first extracted the feature vectors *f*_encoder_(*x*) for each input *x*. These high-dimensional features were then projected into a low-dimensional space using UMAP, which can be formally written as Equation 15:

(15) $$z_{i}=\text{UMAP}(f_{\text{encoder}}(x_{i})),\;z_{i}\in {\mathbb{R}}^{d_{\text{low}}}$$

where *d*_low_ denotes the dimension of the embedding space after UMAP.

The low-dimensional features were subsequently clustered to construct class centers. Let *C_k_*denote the center of cluster *k*; the distance of each sample *i* to its corresponding cluster center is computed as Equation 16:

(16) $$D_{i}=||z_{i}-C_{y_{i}}||2$$

where *y_i_*is the cluster label assigned to sample *i*.

Finally, to quantify the relationship between the encoder representations and segmentation performance, we computed the Pearson correlation coefficient *r* and the associated significance p between the distance *D_i_*and the final test IoU for each sample ${\text{IoU}}_{i}$, the detailed computation is given in Equation 17:

(17) $$r,p=\text{corr}(D_{i},{\text{IoU}}_{i})$$

This analysis allows us to assess how the structural properties of encoder features relate to the final segmentation results, and to evaluate the effectiveness of different modeling approaches in capturing discriminative representations.

## 3 Results and analyses

To validate whether the proposed representation-first modeling strategy generalizes across agricultural scenarios with varying environmental complexity, experiments were conducted under two representative visual conditions. Specifically, segmentation performance was evaluated on datasets with relatively simple backgrounds as well as on datasets characterized by complex and cluttered backgrounds. This experimental design enables a systematic analysis of model behavior across different levels of background complexity, as presented in Sections 3.1 and 3.2.

### 3.1 Performance of leaf lesion segmentation in simple background scenarios

**Figure 4:**
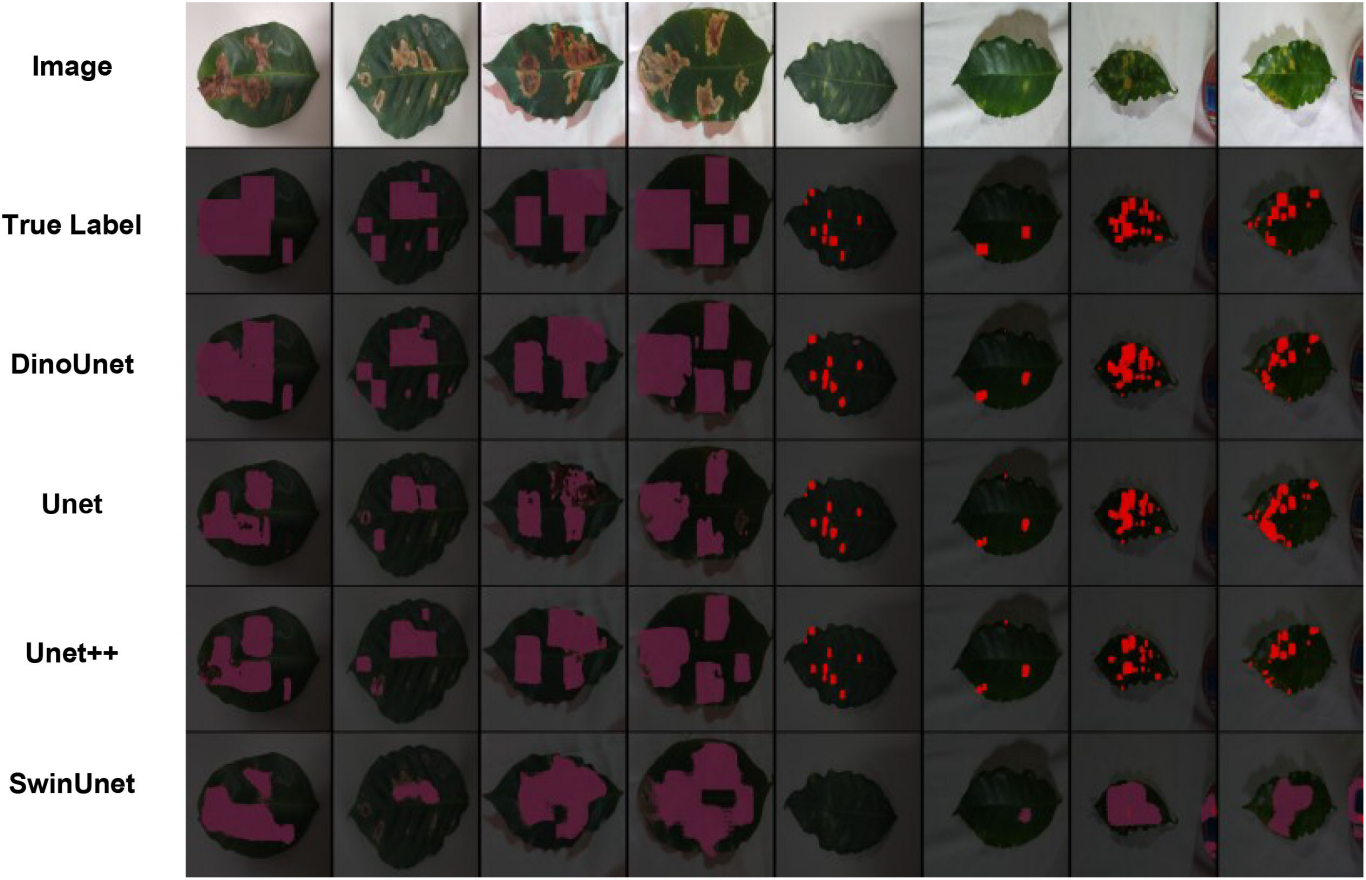
Visualization of coffee leaf damage detection in a laboratory environment.

**Figure 5:**
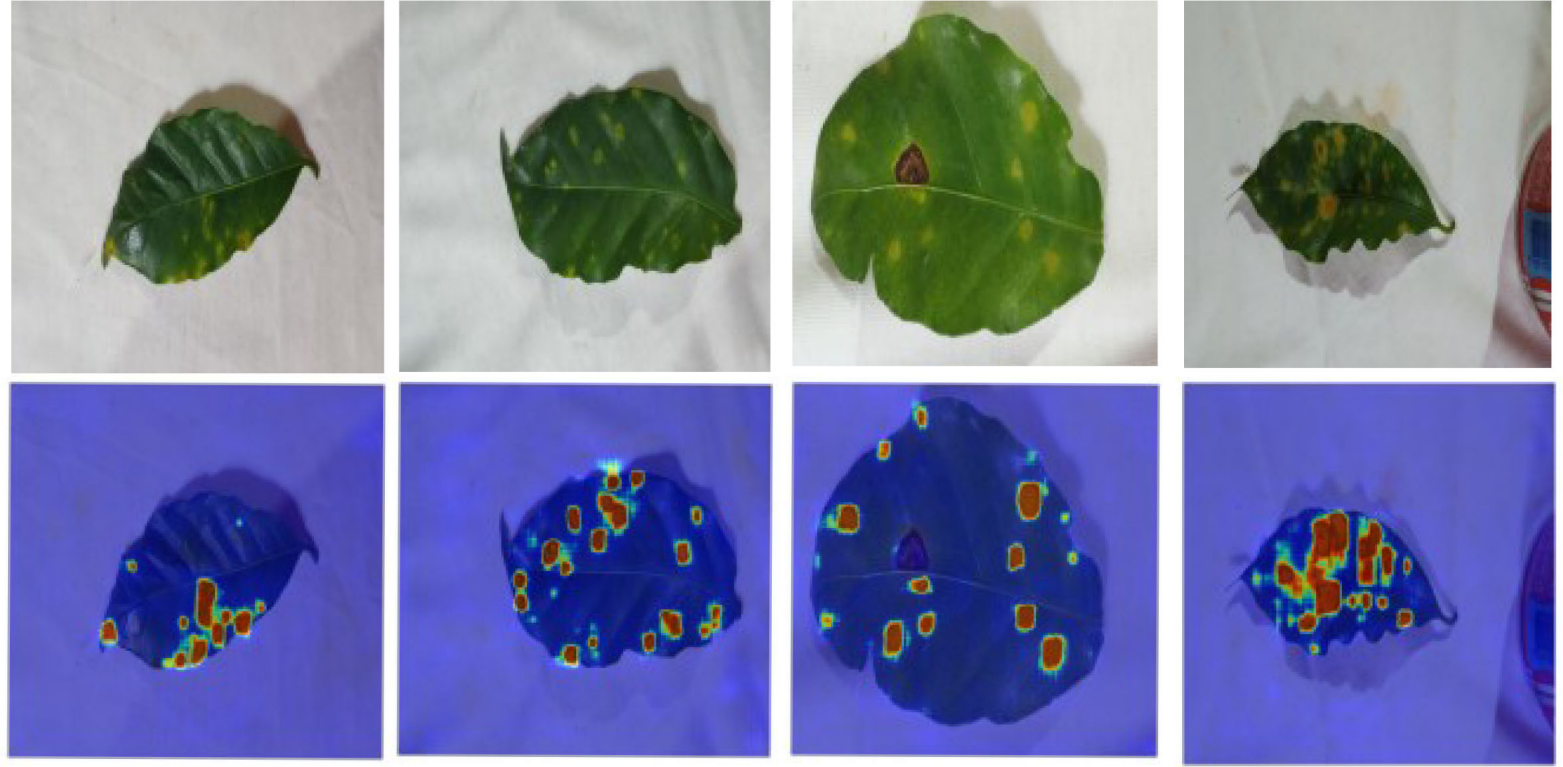
Visualization of attention weights across different model layers.

In this section, we conducted segmentation experiments on two types of coffee leaf lesions collected under controlled laboratory conditions, and visualized the corresponding results, as shown in **Figure 4**. The attention weight maps are shown in **Figure 5**. From a qualitative perspective, DinoUnet and Unet++ exhibit the most favorable segmentation performance. As illustrated in the second column of **Figure 4**, when segmenting fine-grained rust lesions, DinoUnet is able to accurately delineate small and subtle lesion regions, whereas Unet++tends to overlook these minor affected areas. In contrast, Unet and SwinUnet show inferior performance, generally detecting only lesions with prominent visual characteristics while failing to identify smaller or less salient regions. For miner lesion segmentation, SwinUnet does not produce convincing segmentation results. Both Unet and Unet++ demonstrate reasonable performance in this scenario. Although miner lesions do not exhibit visual patterns as salient as rust lesions, their relatively consistent color and shape distributions enable effective learning-based segmentation through supervised training.

**Figure 6:**
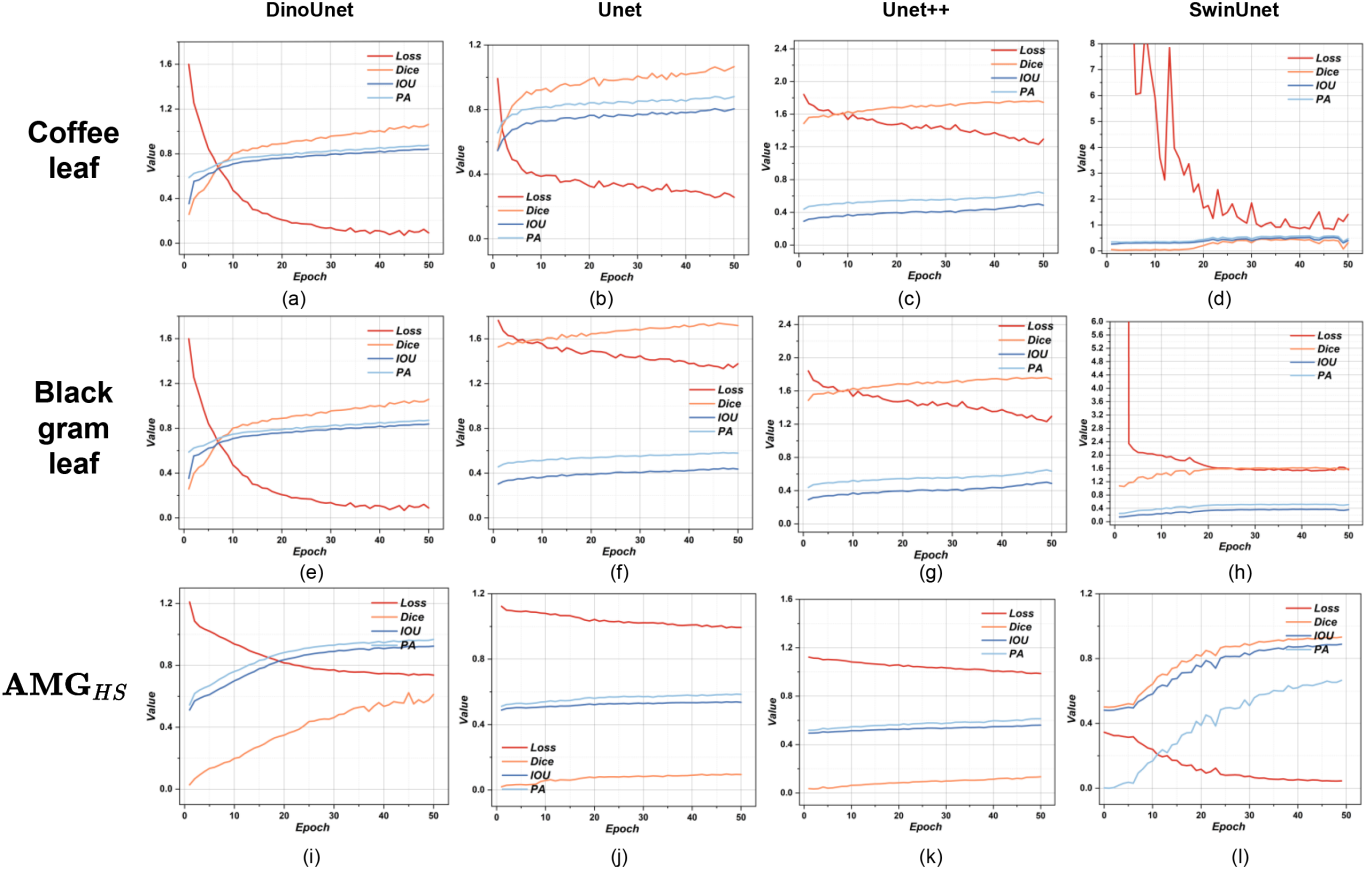
Evolution of training metrics for pest and disease damage detection. **(A–D)** coffee leaf detection; **(E–H)** black gram pest and disease detection; **(I–L)** health and stress detection on AMG*_HS_*.

**Figures 6A–D** illustrates the training dynamics of the four segmentation models in terms of loss convergence and segmentation metrics (Dice, IoU, and pixel accuracy). Distinct differences in convergence behavior and training stability can be observed across models. DinoUnet exhibits rapid and stable convergence during training. The loss decreases sharply within the initial epochs and reaches a low plateau early, while Dice, IoU, and pixel accuracy increase steadily and stabilize after a relatively small number of training epochs. his behavior indicates that DinoUnet is able to achieve effective feature fitting with fewer training iterations, reflecting efficient optimization and strong representation capacity. In contrast, Unet shows a slower convergence trend. Although the loss consistently decreases and segmentation metrics gradually improve, the model requires substantially more epochs to approach a stable performance level, suggesting limited representational efficiency compared to DinoUnet. Unet++ demonstrates a more complex training behavior. While its segmentation metrics increase steadily, the loss remains comparatively high and decreases more slowly throughout training. This indicates that the densely connected decoder structure introduces additional optimization complexity, leading to slower convergence despite reasonable final performance. SwinUnet displays the most unstable training process among all evaluated models. The loss curve fluctuates significantly during early epochs, and segmentation metrics remain low with only marginal improvement over time. Such instability suggests difficulties in optimization and limited effectiveness in learning lesion-specific features under the given training conditions.

**Figure 7:**
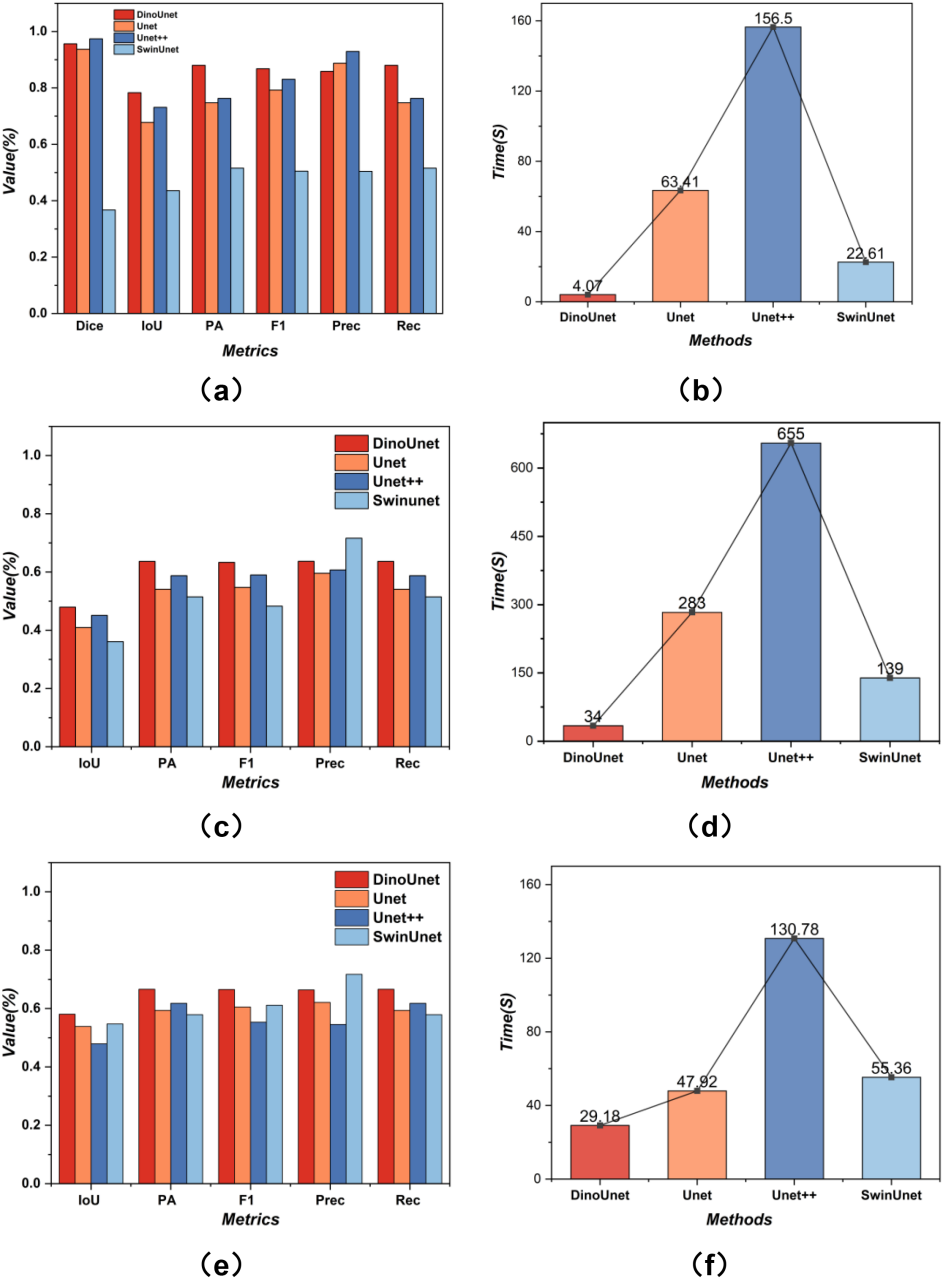
Comparison of detection performance and inference speed on the test set. **(A, B)** coffee leaf pest and disease; **(C, D)** black gram leaf pest and disease; **(E, F)** AMG*_HS_*health and stress detection.

Across all evaluated metrics, as shown in **Figures 7A, B** and **Table 2**, DinoUnet and Unet++ demonstrated consistently strong segmentation performance, substantially outperforming the baseline Unet and SwinUnet models. n particular, Unet++ achieved the highest Dice score (0.974) and precision (0.9292), indicating accurate delineation of lesion regions with limited false positives. DinoUnet exhibited competitive performance, with a Dice score of 0.9562 and balanced precision–recall behavior. In contrast, SwinUnet showed markedly inferior performance across all segmentation metrics, with a Dice score of 0.3672 and IoU of 0.4356, suggesting limited robustness under the evaluated conditions. Pixel accuracy (PA) and F1 scores followed similar trends, further confirming the performance gap between transformer-based and convolutional architectures in this setting. Notably, inference time varied substantially across models. DinoUnet achieved the fastest inference speed (4.07 s), while Unet++ incurred the highest computational cost (156.50 s), highlighting a clear trade-off between segmentation accuracy and efficiency.

**Table 2:** Performance comparison of different models for coffee leaf pest and disease detection.

Method	Dice	IoU	PA	F1	Prec	Rec	Time
DinoUnet	0.9562	0.7831	0.8800	0.8679	0.8586	0.8800	4.07 s
Unet	0.9370	0.6776	0.7471	0.7923	0.8875	0.7471	63.41 s
Unet++	0.9740	0.7309	0.7627	0.8307	0.9292	0.7627	156.50 s
SwinUnet	0.3672	0.4356	0.5156	0.5046	0.5039	0.5156	22.61

From a metric-driven perspective, the superior Dice, IoU, and F1 scores achieved by DinoUnet and Unet++ underscore their enhanced capability to capture complex lesion structures while maintaining spatial consistency. While Unet++ exhibits higher precision—suggesting a more conservative prediction behavior-DinoUnet demonstrates a more balanced precision-recall profile, 379 which is highly desirable for robust performance in practical agricultural monitoring.

**Figure 8:**
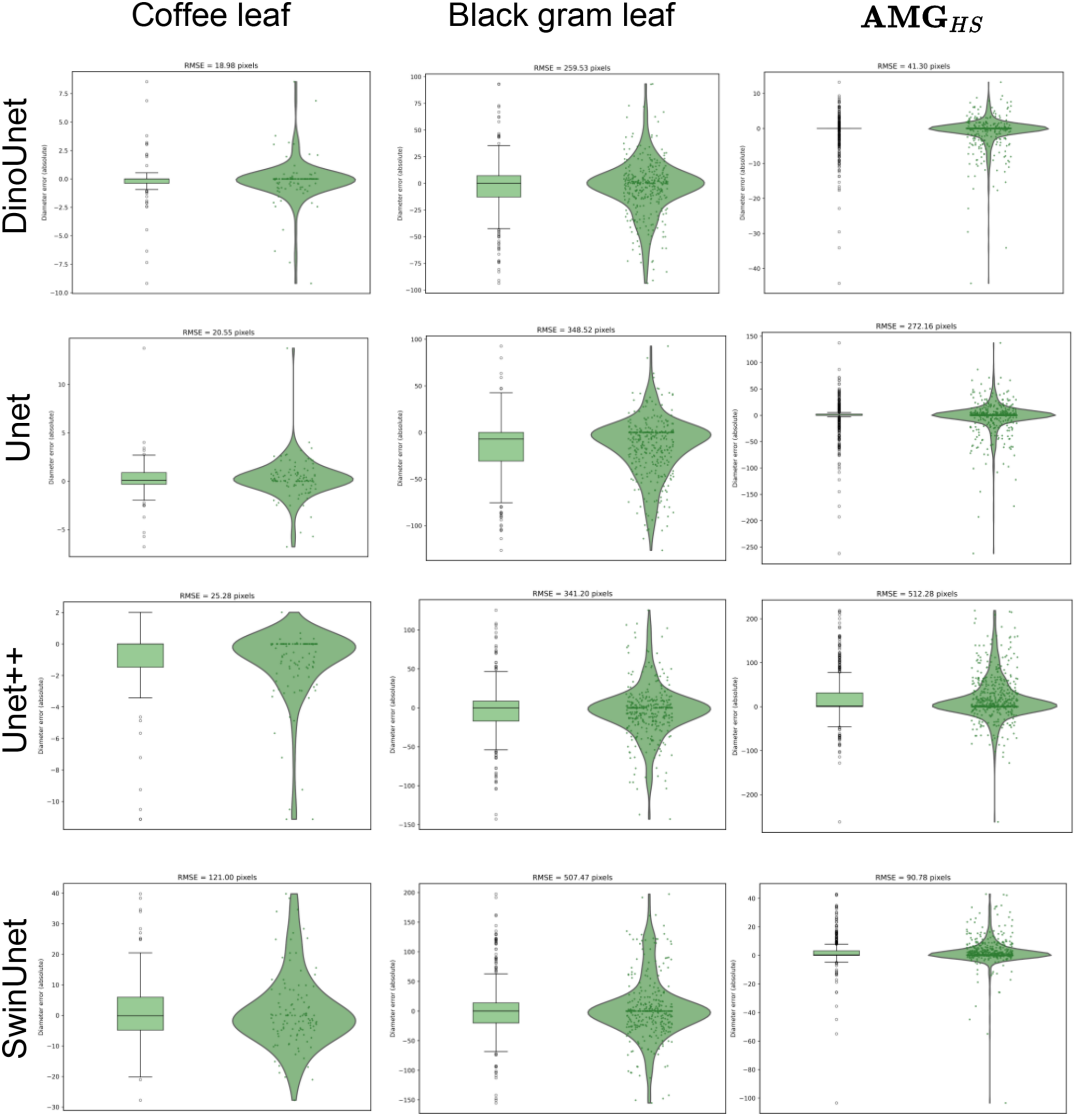
Statistical analysis of diameter measurement accuracy. The plots illustrate the distribution of absolute errors and the Root Mean Square Error (RMSE) for diameter estimations across the test samples.

This macro-level superiority is further validated by micro-level boundary precision. As shown in **Figure 8**. Specifically, DinoUnet achieved the lowest RMSE of 18.98 for lesion circumference, outperforming Unet (20.55) and Unet++ (25.28). This indicates that the semantic richness of foundation-model representations allows for more precise delineation of fine-grained necrotic edges compared to traditional hierarchical aggregations. In stark contrast, SwinUnet exhibited a significantly higher error (RMSE = 121.00) alongside poor segmentation scores. This suggests that transformer-based architectures may struggle to generalize under limited data regimes or when local texture cues dominate lesion appearance. Such observations underscore the critical importance of both representation quality and appropriate inductive bias in advancing precise and reliable agricultural image segmentation.

Importantly, DinoUnet’s strong performance combined with its substantially reduced inference time suggests that a representation-first modeling strategy can effectively balance accuracy and efficiency. Such characteristics are critical for real-world deployment, where large-scale field data and real-time analysis are often required. Despite exhibiting similar segmentation accuracy, DinoUnet outperforms Unet++ when both performance and computational efficiency are jointly considered. The superior efficiency of DinoUnet stems from its Dinov3-based encoder, a foundation model capable of extracting semantically rich and transferable representations, which facilitates fast lesion detection without incurring excessive computational overhead.

### 3.2 Performance of leaf lesion segmentation in complex background scenarios

To further evaluate the robustness of our proposed Representation-First modeling strategy under challenging conditions, we assessed model performance on datasets with complex and cluttered backgrounds. Unlike datasets with simple backgrounds, these images contain multiple visual distractions, such as overlapping leaves, soil textures, and varying illumination, which can substantially impair segmentation accuracy. Under these conditions, the evaluation not only tests the model’s ability to distinguish between lesions and healthy leaf regions, but also emphasizes its capability to accurately handle multiple lesions within the same image.

**Figure 9:**
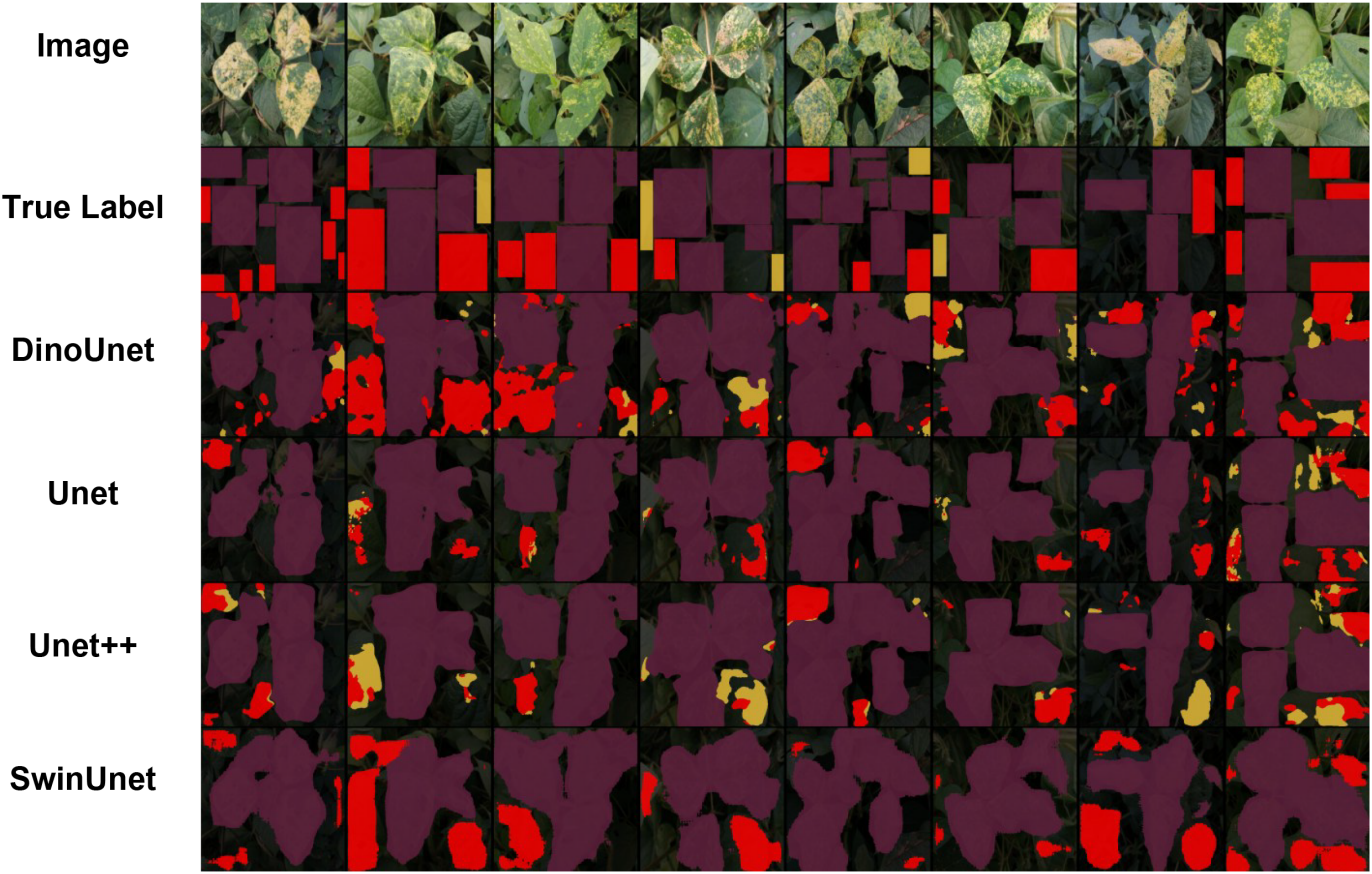
Visualization of black gram leaf damage detection in a field environment.

Under outdoor conditions, all four methods successfully segmented the major Yellow mosaic lesions (purple in **Figure 9**), with DinoUnet most closely matching the ground truth. For healthy leaves (red) and Flea beetle-affected areas (yellow), DinoUnet and SwinUnet generally identified healthy regions correctly but occasionally misclassified them as Flea beetle. In contrast, Unet and Unet++ frequently failed to distinguish healthy leaves from Flea beetle-affected areas. Training dynamics revealed marked architectural disparities in convergence behavior and representational capacity (**Figures 6E–H**). DinoUnet exhibited the most rapid fitting, with its loss function decreasing by 82% within the first 5 epochs and the Dice coefficient concurrently ascending above 0.92. This expedited convergence is attributable to its frozen Dinov3 encoder backbone, which leverages self-supervised pretraining to acquire universal visual representations, thereby obviating the need for decoder parameter training. Specifically, the multi-scale feature extraction mechanism of Dinov3 can be directly transferred to dense prediction tasks, circumventing the prolonged representation learning phase required by conventionally randomly-initialized encoders. In contrast, SwinUnet’s Transformer-based architecture, albeit eventually convergent, demonstrated a characteristically phased loss reduction: a plateau during the initial 15 epochs, followed by gradual optimization driven by sufficient data volume. This lag stems from the self-attention mechanism’s dependency on large-scale data and the representational learning overhead due to the absence of pretrained weights. Notably, despite eventual convergence on the validation set, its performance degraded markedly on the test set, implying an overfitting propensity to training data—likely because the low-inductive-bias nature of Transformers predisposes them to capture dataset-specific noise rather than domain-invariant features. Unet and Unet++ exhibited constrained performance in complex backgrounds, with convergence rates substantially inferior to DinoUnet. This limitation primarily originates from their synchronous encoder-decoder training paradigm: the encoder must learn hierarchical features from scratch while simultaneous decoder optimization destabilizes gradient flow. In cluttered-background scenarios, this process particularly disrupts the progressive alignment of low-level detail and high-level semantic features. Although Unet++’s dense skip connections enhance feature reuse, they also introduce additional trainable parameters and optimization complexity, preventing it from reaching DinoUnet’s epoch-10 performance level even after 25 epochs.

Pretrained representations are pivotal for efficient segmentation: DinoUnet achieves rapid convergence and superior performance via Dinov3 transferability, whereas SwinUnet and Unet variants are constrained by data hunger and synchronous optimization dilemmas, underscoring the indispensability of generic visual features in complex agricultural scenarios.

In complex-background scenarios, the segmentation results highlight pronounced differences among the models (**Table 3**; **Figures 7C, D**). DinoUnet consistently outperformed the other architectures across nearly all metrics: it achieved the highest Dice (0.5754), IoU (0.4793), PA (0.6365), and F1 (0.6332) scores while maintaining a balanced Precision (0.6369) and Recall (0.6365). Notably, its inference time (34s) was substantially lower than Unet (280s) and Unet++ (655s), indicating both efficiency and effectiveness. Unet and Unet++ showed moderate performance but struggled to handle complex backgrounds, reflected in lower IoU, PA, and F1 values. The challenges likely stem from the need to simultaneously learn hierarchical features and decode them in cluttered contexts, where overlapping leaves, soil textures, and multiple lesions disrupt the progressive alignment of low-level and high-level representations. Although Unet++ benefits from dense skip connections, these additional parameters increase optimization difficulty in heterogeneous environments, leading to longer training and inference times without surpassing DinoUnet. SwinUnet, while exhibiting competitive Precision (0.7161), suffered from low Dice (0.5630), IoU (0.3608), PA (0.5145), and F1 (0.4828), indicating that it tends to over-segment or misclassify regions in cluttered scenes. This behavior reflects the Transformer’s dependence on large-scale data and lack of pretraining for domain-specific invariances, which makes it less robust to noisy, highly variable inputs.

**Table 3:** Performance comparison of different models for black gram pest and disease detection.

Method	Dice	IoU	PA	F1	Prec	Rec	Time
DinoUnet	0.5754	0.4793	0.6365	0.6332	0.6369	0.6365	34 s
Unet	0.5256	0.4093	0.5407	0.5473	0.5958	0.5407	280 s
Unet++	0.5852	0.4512	0.5873	0.5898	0.6070	0.5873	655 s
SwinUnet	0.5630	0.3608	0.5145	0.4828	0.7161	0.5145	139 s

To further evaluate the robustness of our representation-first paradigm, we conducted a comparative analysis of lesion boundary precision under both controlled laboratory and complex field environments. As shown in the **Figure 8**, the proposed DinoUnet consistently maintains the highest boundary fidelity, achieving a superior RMSE of 18.98 in the lab and 259.53 in the field. Notably, while transition to field conditions causes a performance decline across all models due to unpredictable background noise and lighting fluctuations, DinoUnet exhibits the most stable degradation profile. In the field environment, it outperforms Unet (348.52) and Unet++ (341.20) by a significant margin, reducing the boundary error by approximately 25.5% and 23.9% respectively. In contrast, the performance of SwinUnet collapses in the field, with its RMSE surging from 121.00 to a staggering 507.47. This drastic increase suggests that without the robust semantic priors provided by a foundation-model encoder, pure transformer-based architectures are highly sensitive to the low-contrast and cluttered textures characteristic of real-world agricultural scenes. These findings confirm that leveraging Dinov3’s pre-trained representations provides a critical advantage in bridging the gap between ideal laboratory research and practical, precise field diagnostics.

The superior performance of DinoUnet in these challenging scenarios underscores the value of foundation models with pretrained visual representations. Its frozen Dinov3 encoder provides robust, general-purpose multi-scale features that are directly transferable to dense prediction tasks, allowing the model to effectively distinguish lesions from healthy regions even under severe background complexity. These results confirm that leveraging foundation models is a promising strategy for agricultural image analysis in real-world, visually complex conditions.

### 3.3 Performance of leaf lesion segmentation in larger-scale datasets

**Figure 10:**
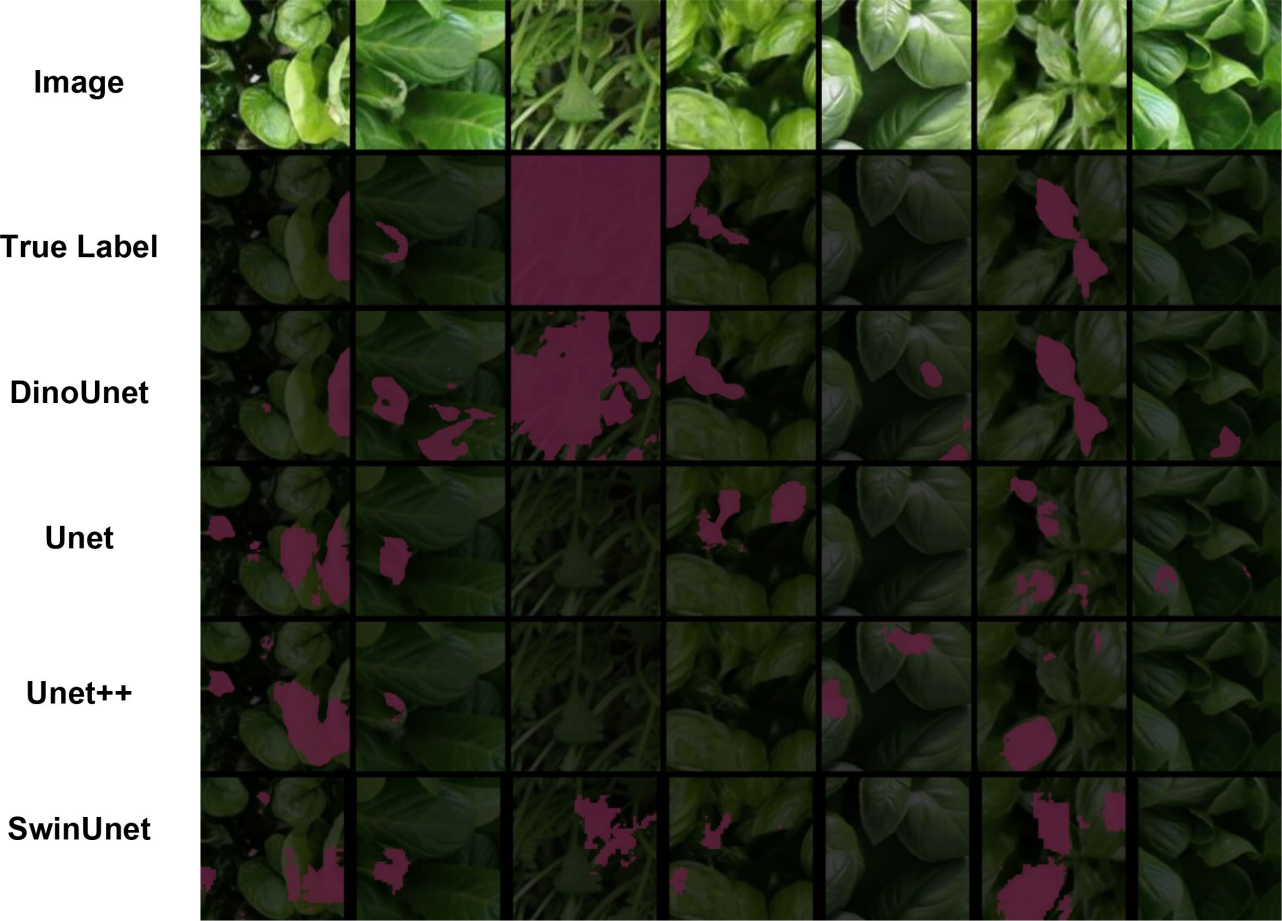
Visualization of leaf damage detection in AMG*_HS_*dataset.

In this subsection, we further validate the effectiveness and stability of the foundation model-based segmentation paradigm for crop disease lesion segmentation on a larger-scale dataset. The segmentation results are shown in **Figure 10**.

As shown in **Table 4** and **Figures 7E, F**, among the five evaluation metrics, DinoUnet achieves the best or second-best segmentation performance in most scenarios. With the expansion of the training data, the overall performance of Transformer-based methods improves significantly. The Precision (Prec) reaches 0.7169. This result indicates that, under large-scale data conditions, the model produces more conservative and accurate predictions for lesion regions. The false positive rate is reduced. The ability to distinguish foreground from background in complex scenes is further enhanced. Compared with conventional segmentation networks trained from scratch, DinoUnet maintains high Precision while still outperforming other models in comprehensive metrics such as Dice and IoU. This finding suggests that the reduction of false detections does not sacrifice the coverage of true lesion regions. The overall segmentation quality is more balanced and robust.

**Table 4:** Performance comparison of different models for health and stress detection on the AMG*_HS_*dataset.

Method	IoU	PA	F1	Prec	Rec	Time
DinoUnet	0.5806	0.6661	0.665	0.6639	0.6661	29.18 s
Unet	0.5384	0.5936	0.605	0.6208	0.5936	47.92 s
Unet++	0.4792	0.6177	0.5531	0.5452	0.6177	130.78 s
SwinUnet	0.5473	0.5789	0.6114	0.7169	0.5789	55.36

In terms of inference efficiency, we conduct batch testing on 1225 images with a resolution of 120 × 120. DinoUnet completes all inference tasks in 29.18 s. For boundary segmentation accuracy, as shown in **Figure 8**, we analyze the fitting ability of different models to lesion boundary scales using the RMSE metric. DinoUnet achieves an RMSE of 41.30 pixels. This value is significantly better than that of SwinUnet (90.78 pixels), Unet (272.16 pixels), and Unet++ (512.28 pixels). These results indicate that the feature representations derived from the foundation model have stronger scale sensitivity and structural preservation capability when modeling lesion boundaries. The model can more accurately reconstruct the true edge morphology. In contrast, traditional convolutional networks tend to produce over-smoothed boundaries or shape deviations when large-scale and small-scale lesions coexist. This limitation leads to significantly increased errors.

In addition, as shown in **Figures 6I–L**, under the condition of a larger dataset and a limited number of training epochs (50 epochs), different models exhibit clear differences in convergence speed. Unet and Unet++ do not fully converge within 50 epochs. This behavior is mainly due to their random initialization and end-to-end training strategy. Their feature extraction process must relearn semantic representations for the downstream task. When data complexity increases, more iterations are required to stabilize the collaborative representation of low-level and high-level 505 features. Therefore, their convergence is relatively slow. In contrast, DinoUnet adopts a pretrained Dino encoder and freezes the encoder during training. Only the lightweight decoder is optimized. This design significantly reduces the number of trainable parameters and the optimization difficulty. The pretrained features already contain strong semantic representations. As a result, the model obtains stable feature distributions in the early training stage. The loss decreases faster and the model converges more quickly. SwinUnet can also converge relatively fast within limited epochs. This advantage is mainly attributed to its hierarchical Transformer architecture and window-based attention mechanism, which improve modeling efficiency in capturing both local and global information. However, due to its larger parameter size and the need for end-to-end training, its final performance and convergence stability remain slightly inferior to DinoUnet with a frozen encoder.

## 4 Discussion

**Figure 11:**
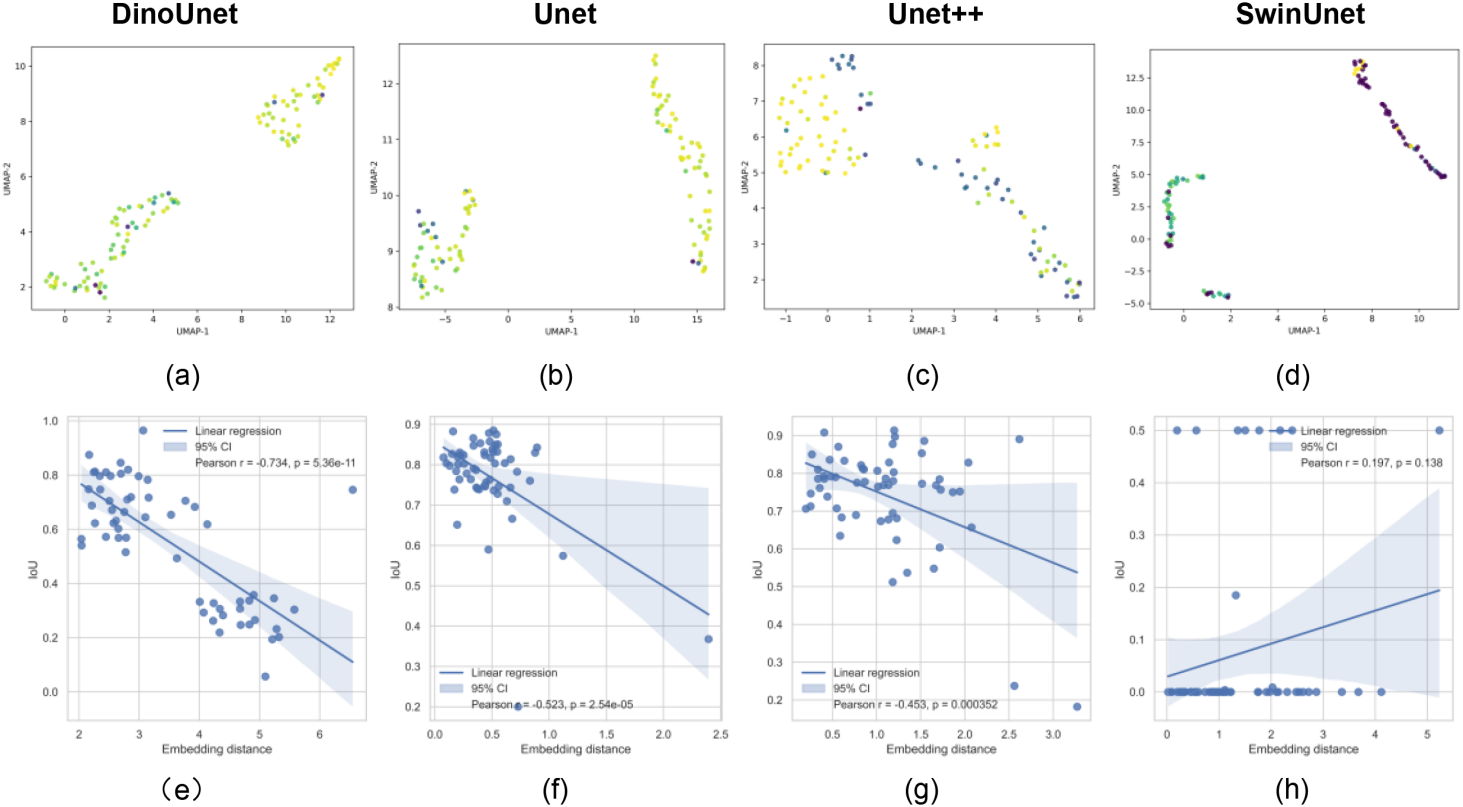
Explainability analysis of the model for coffee leaf damage detection. **(A–D)** Spatial distribution of feature embeddings generated by the encoder; **(E–H)** Correlation analysis between the model’s feature extraction capability and its detection performance.

**Figure 12:**
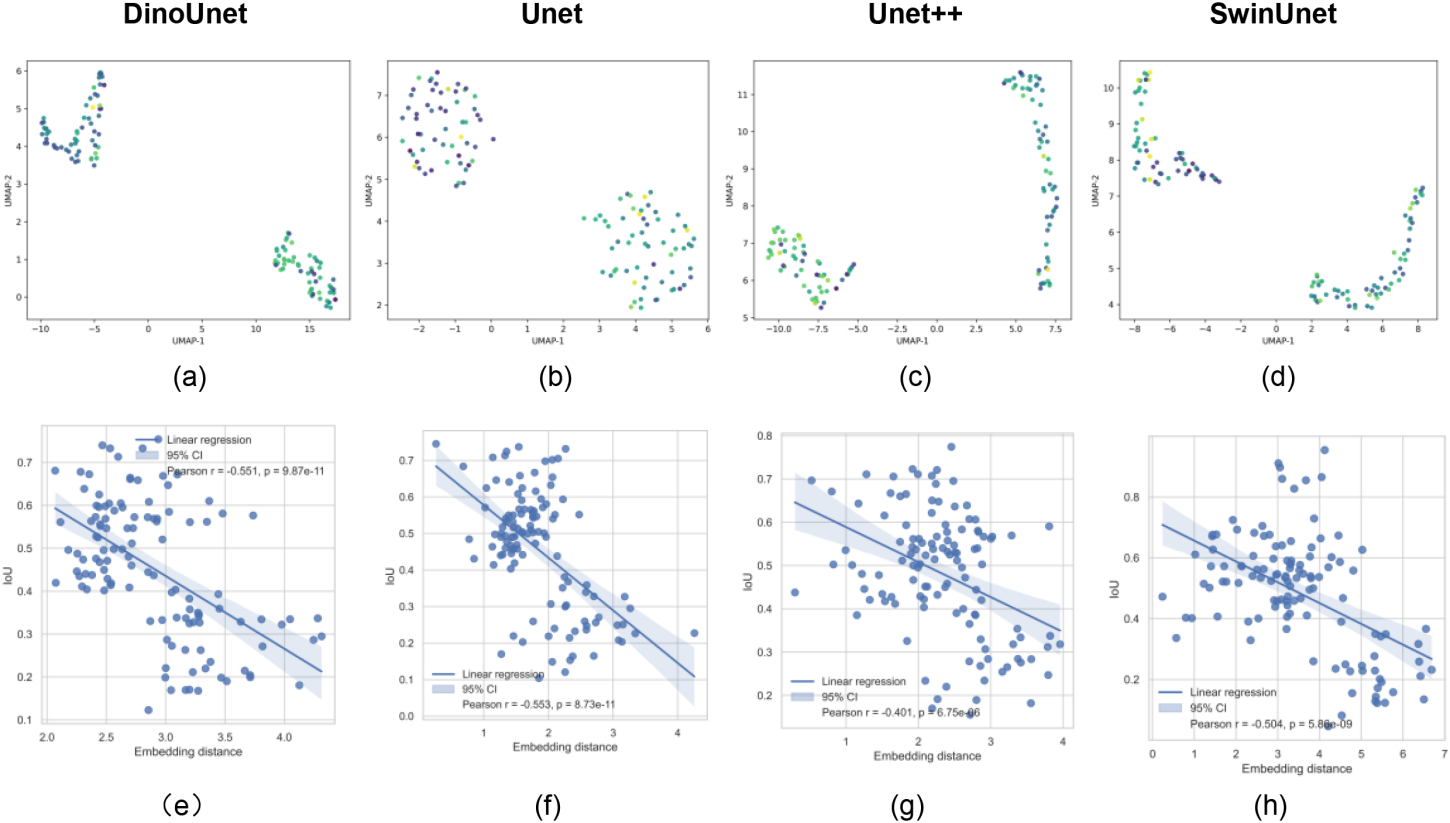
Explainability analysis of the model for black gram leaf damage detection. **(A–D)** Spatial distribution of feature embeddings generated by the encoder; **(E–H)** Correlation analysis between the model’s feature extraction capability and its detection performance.

Although DinoUnet outperforms other models in both simple and complex scenarios, this performance alone does not prove that the advantage derives from the use of foundation models like Dinov3. To probe the source of its robustness, we extracted encoder feature vectors from all methods and visualized them after dimensionality reduction (**Figures 11**, **12**). The corresponding explainability analysis for the AMG*_HS_*dataset is presented in the Appendix.

DinoUnet exhibits tightly clustered intra-class features, reflecting high internal consistency and low variance across similar structures or lesions. Inter-class boundaries are sharply defined, enhancing discriminability and reducing misclassification at object edges. Compared with Unet and Unet++, which show partial cluster overlap, DinoUnet demonstrates minimal cross-class confusion, indicating that its features inherently separate morphologically similar but functionally distinct regions. Moreover, while SwinUnet achieves reasonable separation, DinoUnet preserves cluster compactness and uniform internal structure, suggesting that Dino’s self-supervised pretraining retains hierarchical semantic information beyond local texture cues.

In essence, DinoUnet constructs a feature space with high cohesion and low coupling between classes, providing a robust representational foundation that supports precise segmentation and strong generalization, particularly in visually complex environments.

To further quantify the relationship between feature representations and segmentation performance, we computed the correlation between each sample’s segmentation metrics and its distance to the corresponding class center in the feature embedding space (**Figures 11**, **12**). The Pearson correlation coefficients were consistently concentrated around -0.4 to -0.5, with p-values near zero, indicating a robust negative correlation. This demonstrates that samples whose embeddings lie closer to the class centroid generally achieve higher segmentation accuracy, highlighting the critical role of feature space structure in downstream task performance.

Beyond mere correlation, these results suggest that the model’s ability to organize representations into well-separated, compact clusters is directly linked to reliable segmentation. Embedding quality can thus serve as a predictive indicator of model performance, potentially guiding model selection or hyperparameter tuning. Moreover, these findings support the notion that foundation model-based architectures, such as DinoUnet, provide a generalizable design paradigm: the pre-trained backbone generates embeddings that capture both inter-class discriminability and intra-class cohesion, enhancing robustness to complex visual variability, such as overlapping objects, heterogeneous textures, or lighting fluctuations.

Collectively, this evidence reinforces the value of self-supervised, foundation-model pretraining for dense prediction tasks. By establishing a structured, semantically meaningful feature space, these models not only improve segmentation accuracy but also offer a pathway toward scalable and widely applicable solutions across diverse and visually challenging domains.

From an application perspective in agricultural imaging, DinoUnet demonstrates clear advantages in terms of segmentation accuracy, model complexity, and computational efficiency. In complex field conditions, where leaves overlap, illumination varies, and lesions present heterogeneous textures, DinoUnet achieves higher Dice, IoU, and F1 scores compared with Unet, Unet++, and SwinUnet, reflecting its superior ability to accurately delineate both major and subtle disease regions. The underlying reason is its Dino-pretrained backbone, which produces robust, semantically structured feature embeddings that remain discriminative across diverse visual conditions.

Despite its high performance, DinoUnet maintains a manageable model complexity. Unlike SwinUnet, which relies on Transformer self-attention mechanisms that are data- and compute- intensive, DinoUnet benefits from a frozen backbone, reducing the number of trainable parameters and simplifying optimization. Unet++ introduces dense skip connections, improving feature reuse but increasing optimization difficulty and training time, whereas DinoUnet achieves comparable or better results with fewer epochs.

Efficiency is a critical consideration for large-scale agricultural deployment. DinoUnet achieves inference times far lower than Unet and Unet++, without compromising segmentation quality, making it suitable for real-time or near-real-time monitoring in the field. Importantly, these findings do not suggest that architectural design is unimportant, but rather highlight the central role of representation quality under limited supervision. Taken together, these results suggest that foundation model-based architectures, exemplified by DinoUnet, offer a practical, scalable solution for crop disease monitoring, balancing accuracy, model simplicity, and operational efficiency in real-world agricultural environments.

Recent studies in agricultural segmentation have explored foundation models such as SAM and Dino. However, these models differ in their design philosophy. SAM is a prompt-based model and requires additional inputs such as points or bounding boxes to generate segmentation masks. This increases annotation effort and may limit scalability in large agricultural datasets. In contrast, Dino-based foundation models learn visual representations through self-supervised learning and do not require extra prompts during inference. This makes them more suitable for direct adaptation to downstream segmentation tasks. In the future, box annotations could be used as weak labels, and SAM could be employed as an encoder backbone for segmentation. Such a framework may combine the strong representation ability of SAM with more efficient annotation strategies, which is a promising direction for agricultural image segmentation. An important direction for future work is to establish a standardized evaluation framework for different foundation models. Under unified data splits, consistent training strategies, and aligned evaluation protocols, we will conduct systematic comparative and ablation analyses across representative encoders, including DINOv3, DINOv2, MAE, and SAM. This extension will enable a clearer assessment of how different pre-training paradigms affect representation quality and downstream segmentation performance in agricultural scenarios. A unified and controlled setting will also help ensure fair comparison and support more reliable conclusions regarding model selection and practical deployment.

YOLO models are mainly designed for real-time object detection. They use a single-stage, end-to-end framework to predict bounding boxes and class probabilities. Their core design focuses on detection efficiency and fast deployment under limited computational cost. Although some YOLO variants support instance or semantic segmentation, segmentation is usually an auxiliary branch rather than the main objective. In contrast, our study does not aim to propose a new SOTA architecture through complex structural design. Instead, we show that combining a pretrained foundation model with a simple decoder can improve representation ability while keeping the model efficient and structurally simple. This approach uses large-scale pretrained knowledge to achieve accurate and robust segmentation without heavily engineered task-specific modules. Our results suggest that pairing strong foundation models with lightweight downstream heads is an effective and scalable strategy. In future work, we will explore integrating foundation models with YOLO-style architectures to better combine detection-oriented frameworks with global pretrained representations for unified detection and segmentation tasks.

## 5 Conclusion

In summary, this study demonstrates that leveraging foundation model-based representations substantially enhances segmentation robustness in agricultural imagery, particularly when navigating the transition from controlled settings to complex, unstructured field conditions. Through a synthesis of qualitative visualizations, quantitative metrics (including boundary RMSE), and feature-space interpretability, we establish that superior segmentation efficacy is anchored by the richness of learned representations rather than architectural complexity alone. The proposed DinoUnet consistently achieves state-of-the-art accuracy across multiple species while delivering a 93.6% reduction in inference time, demonstrating rock-solid reliability for deployment on resource-constrained edge devices. Importantly, these findings do not negate the value of architectural refinement; instead, they emphasize that under limited supervision, high-quality pretrained features serve as the unshakeable cornerstone for generalizable agricultural vision. These insights support the DinoUnet framework as a high-performance, scalable, and unified representation-first paradigm for advancing precise crop health monitoring and automated disease diagnostics.

## Data Availability

Publicly available datasets were analyzed in this study. This data can be found here: https://data.mendeley.com/datasets/vfxf4trtcg/5; https://data.mendeley.com/datasets/45djgf3p96/1.
