# Supplementary material for: Precise leaf damage detection across diverse species and environments via a large-scale vision model
Source: Front Plant Sci. 2026 Mar 26;17:1788926. doi: 10.3389/fpls.2026.1788926 (PMC13062211; doi:10.3389/fpls.2026.1788926)
Supplement: Supplementary file 1 [file Supplementaryfile1.pdf]

Supplementary Material

1 METHODS

1.1 Project Module

The proposed module processes each feature map C'_i through two parallel branches and projects it into a shared intermediate space of rank R. First, a shared 1×1 convolution W_{ctx} extracts a scale-invariant contextual feature $Z_{ctx,i}$. Second, a scale-specific 1×1 convolution $W_{sp,i}$ generates a scale-dependent feature $Z_{sp,i}$ (Eq. (S1)):

$$Z_{ctx,i} = W_{ctx}^T C'_i, \quad Z_{sp,i} = W_{sp,i}^T C'_i. \tag{S1}$$

Instead of concatenation, the contextual feature modulates the scale-specific feature. A small generator $\mathcal{G}_i$ predicts modulation parameters (γ_i, β_i) from $Z_{ctx,i}$ (Eq. (S2)). Feature-wise linear modulation is then applied (Eq. (S3)):

$$(\gamma_i, \beta_i) = \mathcal{G}_i(Z_{ctx,i}), \tag{S2}$$

$$Z_{mod,i} = \gamma_i \odot Z_{sp,i} + \beta_i. \tag{S3}$$

The modulated feature $Z_{mod,i}$ is refined and mapped to the decoder channel dimension D'_i. A scale-specific 1×1 convolution $W_{r,i}$ first produces Y_i, followed by a depthwise separable convolution (Eq. (S4)):

$$Y_i = W_{r,i}^T Z_{mod,i}, \quad Y'_i = \mathcal{C}_{\text{dwsep}}(Y_i). \tag{S4}$$

To enhance channel discrimination, a squeeze-and-excitation (SE) operation is applied. Global average pooling and a two-layer MLP generate a channel attention vector s_i (Eq. (S5)):

$$s_i = \sigma(\text{MLP}(\text{GAP}(Y'_i))). \tag{S5}$$

The refined feature is recalibrated by channel-wise multiplication. Finally, a residual shortcut $\mathcal{P}_i$ ensures stable training and dimension alignment. The final output for each scale is (Eq. (S6)):

$$S_i = (Y'_i \odot s_i) + \mathcal{P}_i(Z_{mod,i}). \tag{S6}$$

The resulting feature maps $\{S_i\}_{i=1}^N$ are used as skip connections for the U-Net decoder.

1.2 Detailed Model Configurations

The default implementation details of the SPM module are as follows. The ConvBlocks consist of a 3×3 convolutional layer (stride 1, padding 1), a BatchNorm2d layer (momentum 0.1, eps=1e-5), and a ReLU activation function (inplace=True). The MaxPooling layer uses a 2×2 kernel with stride 2 and no padding. The overall execution flow of the SPM module is described as follows. The input feature map passes through Conv2d, BatchNorm2d, ReLU, and MaxPooling sequentially. The final output is a downsampled feature map.

For a fair comparison, Unet, Unet++, and SwinUnet are trained under the same experimental settings. The initial learning rate is set to 0.0005. The optimizer is Adam. The weight decay is set to 1×10^{-4}. All models are trained for 50 epochs. The batch size is kept the same across all experiments. The loss function is the combination of Cross-Entropy loss and Focal loss with equal weights. All models are trained end-to-end using random initialization, except for DinoUnet, which adopts a pretrained Dino encoder with frozen parameters during training. Only the decoder part of DinoUnet is optimized.

1.3 Diameter-based Root Mean Square Error

To quantitatively evaluate morphological accuracy, we compute the equivalent diameter of each segmented region.

For a binary mask $M \in \{0,1\}^{H \times W}$, its area is defined as (Eq. (S7)):

$$A = \sum_{x=1}^{H} \sum_{y=1}^{W} M(x,y). \tag{S7}$$

The equivalent diameter is defined as the diameter of a circle with identical area (Eq. (S8)):

$$D = 2\sqrt{\frac{A}{\pi}}. \tag{S8}$$

For the i-th sample and class c, let $A_{i,c}^{pred}$ and $A_{i,c}^{gt}$ denote the predicted and ground-truth areas, respectively. Their corresponding equivalent diameters are (Eqs. (S9), (S10)):

$$D_{i,c}^{pred} = 2\sqrt{\frac{A_{i,c}^{pred}}{\pi}}, \tag{S9}$$

$$D_{i,c}^{gt} = 2\sqrt{\frac{A_{i,c}^{gt}}{\pi}}. \tag{S10}$$

The diameter error is defined as (Eq. (S11)):

$$e_{i,c} = D_{i,c}^{pred} - D_{i,c}^{gt}. \tag{S11}$$

For each class c, we compute the root mean square error (RMSE) only over samples whose ground-truth area is non-zero (Eq. (S12)):

$$\text{RMSE}_c = \sqrt{\frac{1}{N_c} \sum_{i=1}^{N_c} (e_{i,c})^2}, \tag{S12}$$

where N_c is the number of valid samples satisfying $A_{i,c}^{gt} > 0$.

The overall RMSE is computed across all valid class–sample pairs (Eq. (S13)):

	coffee leaf			black gram			AMG_{HS}		
	Mean	Variance	Skewness	Mean	Variance	Skewness	Mean	Variance	Skewness
DinoUnet	-1.05	110.43	0.58	8.54	6952.83	0.73	-4.29	1027.61	-1.54
Unet	9.37	5237.65	1.63	-39.81	9218.41	0.08	4.85	8829.43	-1.03
Unet++	-7.99	265.5	-1.71	-10.43	9014.59	-0.08	71.61	24590.36	0.19
SwinUnet	5.73	9653.01	0.45	-22.7	19364.73	-0.39	28.72	2476.07	1.13

Table S1. The quantitative evaluation metric of RMSE

$$\mathrm{RMSE}_{overall} = \sqrt{\frac{1}{N_{total}} \sum_{c=1}^{C} \sum_{i=1}^{N_c} \left(e_{i,c}\right)^2}, \tag{S13}$$

where $N_{total} = \sum_{c=1}^{C} N_c$ denotes the total number of valid class–sample pairs.

Only samples with non-zero ground-truth area are included to avoid artificially inflating the metric for absent objects. This metric primarily measures scale deviation rather than shape similarity.

2 RESULTS

2.1 Quantitative analysis of parameter costs

The core difference between our decoder and the classical U-Net lies in the introduction of a "shared + specific" hybrid convolutional branch: the shared branch enables information propagation across decoder layers of different scales, while the specific branch, through an independent PM module, adapts the high-dimensional features from the pretrained backbone and performs targeted decoding. This design breaks the single decoding logic of classical UNet, which relies solely on same-resolution skip connections. The parameter analysis shows that the additional shared branch and PM module introduce only 1.05 M parameters, accounting for 16.8% of the total parameters of the classical UNet decoder (6.27 M). This demonstrates that our design improves feature representation capability while incurring a controlled and modest increase in parameter cost.

2.2 Feature Visualization and Metric Correlation Results for the AMG_{HS} Dataset

The Fig. S1 illustrates the feature visualization results of different models on the AMG_{HS} dataset, as well as the correlation between feature quality and detection metrics. Similar to the results observed on the Black Gram and Coffee Leaf datasets, it can be clearly seen that better encoder quality leads to higher detection performance.

2.3 The quantitative evaluation metric of RMSE

As shown in Table S1, the mean, variance, and skewness of RMSE for different models across multiple datasets are reported.

The data show that DinoUnet achieves the lowest mean error (-1.05) on the coffee leaf dataset. In contrast, Unet++ shows the highest mean error (71.61) on the AMG_{HS} dataset. In terms of variance, SwinUnet has the largest variance (19364.73) on the black gram dataset, which indicates large fluctuations in its predictions on this dataset.

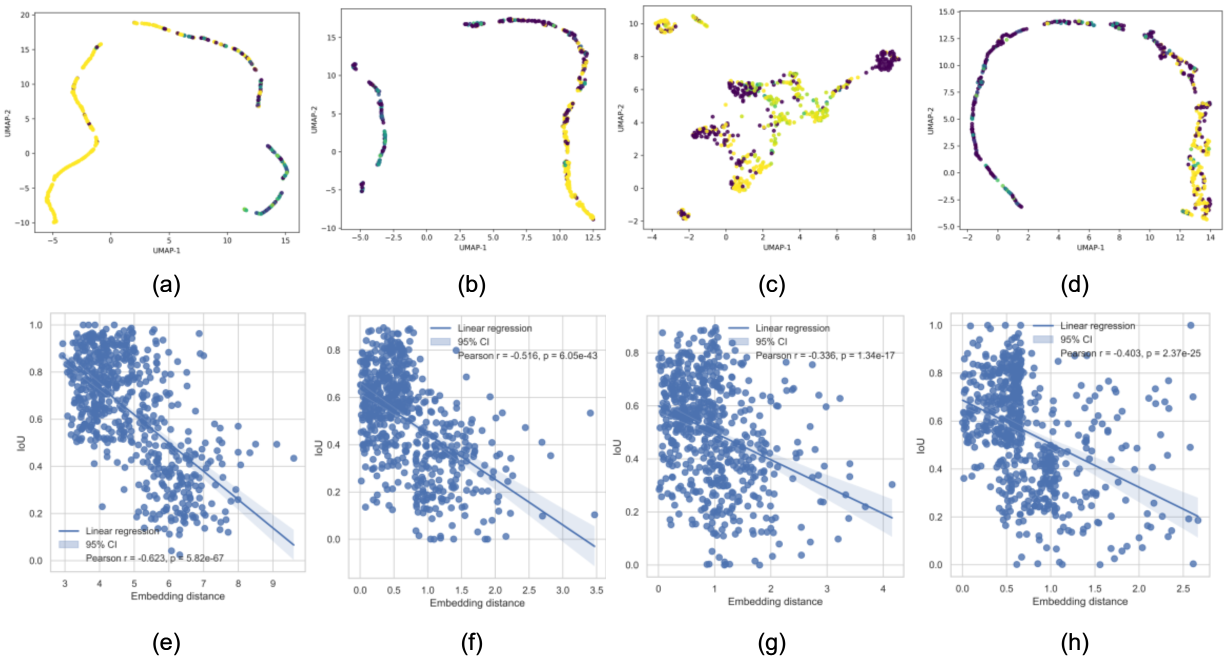

Figure S1: Explainability analysis of the model for AMG$_{HS}$ dataset. (a–d) Spatial distribution of feature embeddings generated by the encoder; (e–h) Correlation analysis between the model's feature extraction capability and its detection performance.

Further differences can be observed across datasets. On the coffee leaf dataset, DinoUnet (-1.05) and Unet++ (-7.99) have negative mean errors, while Unet (9.37) and SwinUnet (5.73) have positive mean errors. This indicates different directions of systematic prediction bias. Unet shows the largest variance (5237.65) on this dataset, which is much higher than DinoUnet (110.43), suggesting lower prediction stability.

On the black gram dataset, the mean errors differ significantly among models. DinoUnet has a mean error of 8.54, while SwinUnet reaches -22.7. The variances are generally high on this dataset. SwinUnet reaches 19364.73, and Unet++ and Unet reach 9014.59 and 9218.41, respectively. Only DinoUnet maintains a relatively lower variance (6952.83), indicating better robustness.

The AMG_{HS} dataset shows more dispersed results. Unet++ has a very high mean error of 71.61 and an extremely large variance of 24590.36. This is the highest variance among all models and datasets. It indicates serious prediction instability. DinoUnet maintains the smallest absolute mean error (-4.29) on this dataset. However, its skewness is -1.54, which indicates a clear left-skewed distribution. SwinUnet shows a much lower variance (2476.07) than other models, but its mean error (28.72) remains relatively high.

From the skewness perspective, Unet++ shows the strongest right skew (1.63) on the coffee leaf dataset. DinoUnet shows the strongest left skew (-1.54) on the AMG_{HS} dataset. Most models have skewness values with absolute values less than 1. This suggests that the RMSE distributions are relatively symmetric. However, some models still show clear skewness on specific datasets. This indicates that the distribution pattern of prediction errors should be considered in practical model evaluation.
